# A machine learning based approach to identify carotid subclinical atherosclerosis endotypes

**DOI:** 10.1093/cvr/cvad106

**Published:** 2023-07-21

**Authors:** Qiao Sen Chen, Otto Bergman, Louise Ziegler, Damiano Baldassarre, Fabrizio Veglia, Elena Tremoli, Rona J Strawbridge, Antonio Gallo, Matteo Pirro, Andries J Smit, Sudhir Kurl, Kai Savonen, Lars Lind, Per Eriksson, Bruna Gigante

**Affiliations:** Division of Cardiovascular Medicine, Department of Medicine Solna, Karolinska Institutet, Solnavägen 30, 171 64 Stockholm, Sweden; Division of Cardiovascular Medicine, Department of Medicine Solna, Karolinska Institutet, Solnavägen 30, 171 64 Stockholm, Sweden; Division of Medicine and Department of Clinical Sciences, Danderyd Hospital, Karolinska Institutet, Entrevägen 2, 182 88 Stockholm, Sweden; Department of Medical Biotechnology and Translational Medicine, Università di Milano, Via Vanvitelli 32, 20133 Milan, Italy; Centro Cardiologico Monzino, IRCCS, Via Carlo Parea 4, 20138 Milan, Italy; Maria Cecilia Hospital, GVM Care & Research, Via Corriera 1, 48033 Cotignola (RA), Italy; Maria Cecilia Hospital, GVM Care & Research, Via Corriera 1, 48033 Cotignola (RA), Italy; Division of Cardiovascular Medicine, Department of Medicine Solna, Karolinska Institutet, Solnavägen 30, 171 64 Stockholm, Sweden; Institute of Health and Wellbeing, University of Glasgow, Clarice Pears Building, 90 Byres Road, Glasgow G12 8TB, UK; Health Data Research, Clarice Pears Building, 90 Byres Road, Glasgow G12 8TB, UK; Lipidology and Cardiovascular Prevention Unit, Department of Nutrition, Sorbonne Université, INSERM UMR1166, APHP, Hôpital Pitié-Salpètriêre, 47 Boulevard de l´Hopital, 75013 Paris, France; Internal Medicine, Angiology and Arteriosclerosis Diseases, Department of Medicine, University of Perugia, Piazzale Menghini 1, 06129 Perugia, Italy; Department of Medicine, University Medical Center Groningen, Groningen & Isala Clinics Zwolle, Dokter Spanjaardweg 29B, 8025 BT Groningen, the Netherlands; Institute of Public Health and Clinical Nutrition, University of Eastern Finland, Kuopio Campus, Yliopistonranta 1 C, Canthia Building, B Wing, FI-70211 Kuopio, Finland; Kuopio Research Institute of Exercise Medicine, Haapaniementie 16, FI-70100 Kuopio, Finland; Department of Clinical Physiology and Nuclear Medicine, Science Service Center, Kuopio University Hospital, Yliopsistonranta 1F, FI-70211 Kuopio, Finland; Department of Medical Sciences, Uppsala University, Uppsala Science Park, Dag Hammarskjöldsv 10B, 752 37 Uppsala, Sweden; Division of Cardiovascular Medicine, Department of Medicine Solna, Karolinska Institutet, Solnavägen 30, 171 64 Stockholm, Sweden; Division of Cardiovascular Medicine, Department of Medicine Solna, Karolinska Institutet, Solnavägen 30, 171 64 Stockholm, Sweden; Department of Cardiology, Danderyd University Hospital, Entrevägen 2, 182 88 Stockholm, Sweden

**Keywords:** Atherosclerosis, Endotype, Artificial intelligence, Progression of atherosclerosis, ASCVD, Biological markers

## Abstract

**Aims:**

To define endotypes of carotid subclinical atherosclerosis.

**Methods and results:**

We integrated demographic, clinical, and molecular data (*n* = 124) with ultrasonographic carotid measurements from study participants in the IMPROVE cohort (*n* = 3340). We applied a neural network algorithm and hierarchical clustering to identify carotid atherosclerosis endotypes. A measure of carotid subclinical atherosclerosis, the c-IMT_mean-max_, was used to extract atherosclerosis-related features and SHapley Additive exPlanations (SHAP) to reveal endotypes. The association of endotypes with carotid ultrasonographic measurements at baseline, after 30 months, and with the 3-year atherosclerotic cardiovascular disease (ASCVD) risk was estimated by linear (*β*, SE) and Cox [hazard ratio (HR), 95% confidence interval (CI)] regression models. Crude estimates were adjusted by common cardiovascular risk factors, and baseline ultrasonographic measures. Improvement in ASCVD risk prediction was evaluated by *C*-statistic and by net reclassification improvement with reference to SCORE2, c-IMT_mean-max_, and presence of carotid plaques. An ensemble stacking model was used to predict endotypes in an independent validation cohort, the PIVUS (*n* = 1061). We identified four endotypes able to differentiate carotid atherosclerosis risk profiles from mild (endotype 1) to severe (endotype 4). SHAP identified endotype-shared variables (age, biological sex, and systolic blood pressure) and endotype-specific biomarkers. In the IMPROVE, as compared to endotype 1, endotype 4 associated with the thickest c-IMT at baseline (*β*, SE) 0.36 (0.014), the highest number of plaques 1.65 (0.075), the fastest c-IMT progression 0.06 (0.013), and the highest ASCVD risk (HR, 95% CI) (1.95, 1.18–3.23). Baseline and progression measures of carotid subclinical atherosclerosis and ASCVD risk were associated with the predicted endotypes in the PIVUS. Endotypes consistently improved measures of ASCVD risk discrimination and reclassification in both study populations.

**Conclusions:**

We report four replicable subclinical carotid atherosclerosis—endotypes associated with progression of atherosclerosis and ASCVD risk in two independent populations. Our approach based on endotypes can be applied for precision medicine in ASCVD prevention.

## Graphical Abstract

**Graphical Abstract:**
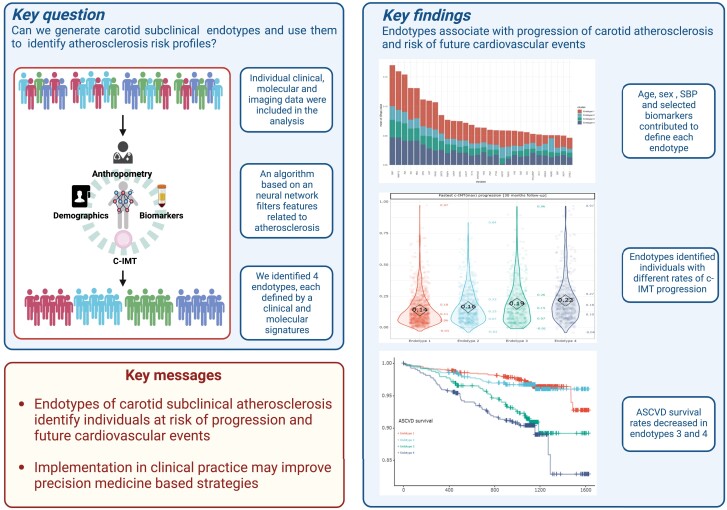

**Time of primary review: 36 days**

## 1. Introduction

Atherosclerotic cardiovascular disease (ASCVD), namely coronary heart disease (CHD) and ischaemic stroke, is the leading cause of premature mortality and disability in Europe.^1^ Heterogeneity characterizes both ASCVD pathophysiology and clinical manifestations and represents the major challenge for implementation of precision medicine.

Deep phenotyping by endotypes is a modern approach to identify molecular signatures underlying selective traits in complex diseases.^2^ Through the integration of demographic, clinical, molecular, and imaging data, endotypes reclassify diseases according to pathophysiological traits and possibly disclose clinical phenotypes and response to therapeutics.^3–5^ Endotyping aims to overcome the limitations of the reductionism that informs clinical practice, where all patients are deemed to respond to a standardized treatment. As most of the novel cardiovascular (CV) drugs are small molecules^6^ or monoclonal antibodies designed to target specific molecular pathways,^7,8^ they are likely to be effective in patients with selected clinical characteristics. Endotypes may provide a solid basis to identify patients amenable for treatment with biological drugs.

Measures of carotid subclinical atherosclerosis may be used to estimate the individual atherosclerosis burden. The carotid intima-media thickness (c-IMT) and the presence of carotid plaque,^9^ the c-IMT progression,^10,11^ and the inter-adventitial common carotid artery diameter (ICCAD)^12^ have been associated with the risk for ASCVD.^9–11^ More recently, a meta-analysis of 119 clinical trials has shown reduced progression of c-IMT associated with a lower ASCVD risk.^13^ Therefore, understanding the pathobiological profiles underlying the dynamic of the progression and regression of carotid atherosclerosis may be of use to timely intervene and reduce the risk of future ASCVD. However, to the best of our knowledge, we still do not have the tools to identify, in the early stages of the disease, those individuals prone to atherosclerosis progression. Endotypes of subclinical atherosclerosis could be used to fill this gap in knowledge.

To this end, we have applied a machine learning approach to define endotypes of carotid subclinical atherosclerosis in a large European cohort, the IMPROVE.^14^ We have estimated the association of endotypes with baseline and progression carotid ultrasonographic measures and with risk of future ASCVD. Further, we have and validated our findings in an independent cohort, the PIVUS. As a secondary aim, we have tested the ability of endotypes to improve ASCVD risk discrimination and reclassification measures.

## 2. Methods

### 2.1 Discovery cohort

The IMPROVE study has been previously described.^10,14^ Briefly, it comprises 3711 study participants (age 55–79, women 52.1%, enrolled during 2004–2005) recruited in five European countries (Italy, France, the Netherlands, Sweden, and Finland). All eligible participants had at least three CV risk factors (i.e. male sex or female sex in women at least 5 years after menopause, dyslipidaemia, hypertension, diabetes, smoking, and family history of ASCVD) at inclusion but not an overt ASCVD or any conditions risking longevity or c-IMT visualization at baseline. Study participants completed a questionnaire on lifestyle habits, previous and current diseases, and medications and underwent a physical examination including ultrasonography of the carotid arteries at baseline. Blood samples were collected and stored at −80°C until analysis. In the present study, we included cardiovascular-related demographic, anthropometric, and clinical variables as well as circulating biomarkers (*n* = 124) measured in 3121 IMPROVE study participants without any missing data (derived dataset). Supplementary material online, *Table S1* lists the variables included in the analysis. Biomarkers were measured in plasma using Olink cardiovascular panel I (CVD-I)^15^ as well as other conventional methods (e.g. enzyme-linked immunosorbent assay) detailed in Supplementary material online, *Table S1*. Two-hundred-nineteen participants with at least one missing value in variables reported in Supplementary material online, *Table S1* were included in a replicated dataset. Supplementary material online, *Figure S1* summarizes the inclusion and exclusion criteria. Study participants were followed up, for incident ASCVD [myocardial infarction (MI), sudden cardiac death, angina pectoris, ischaemic stroke, transient ischaemic attack, and new diagnosis of intermittent claudication or any surgical intervention or revascularization of coronary or peripheral arteries], death or end-of follow-up whichever came first during 36 months at each participating centre.^10,16^ As previously reported,^12^ occurrence of ASCVD was assessed at each participating centre. Events were then centrally adjudicated by a designated specialist (Prof. Ulf de Faire, Karolinska Institutet) who was unaware of clinical history and c-IMT data.

### 2.2 Validation cohort

The Prospective Study of the Vasculature in Uppsala Seniors (PIVUS) comprises 1016 randomly selected 70 years old living in Uppsala (Sweden) (50% female, 50% response rate) enrolled between 2001 and 2004.^17^ Demographic, anthropometric, clinical, and carotid ultrasonographic measures were collected for each study participant. Biomarkers were measured by Olink CVD-I. Data on prevalent and incident fatal/non-fatal MI and ischaemic stroke were collected from the Swedish Hospital Discharge Register. MI was defined by the following diagnosis codes [International Classification of Diseases (ICD)-8 code 410, ICD-9 code 410, or ICD-10 code I20] and ischaemic stroke by ICD-8 codes 431 and 433–436, ICD-9 codes 431 and 433–436, or ICD-10 codes I63 and I66. Study participants with missing values were excluded, leaving 960 participants for analysis.

### 2.3 Ultrasonographic measurements in IMPROVE and PIVUS

A summary of the ultrasonographic protocols followed in IMPROVE and PIVUS has been previously reported.^18^ C-IMT in IMPROVE was measured at four consecutive segments at the far wall of the left and right carotid arteries and reported in millimetres (mm). Data were averaged to estimate the c-IMT_mean_, c-IMT_max_, and c-IMT_mean-max_, i.e. the average of c-IMT_max_ measured at each segment of the whole carotid tree. Carotid plaques, defined as c-IMT ≥ 1.5 mm, were included in the measurement. The total number of plaques was annotated, and the area of the plaque (mm^2^), including (Area of plaques_bulb_) and excluding the carotid bulb (Area of plaques_no-bulb_), was calculated. ICCAD (mm) was measured distally to the bifurcation (2 cm) on both the left and the right carotid arteries in a carotid plaque-free area. In the present study, we included ultrasonographic progression measures after 30 months of follow-up (mm/year): the c-IMT_max-prog_, c-IMT_mean-max-progr_, and c-IMT_fastest-progr_, which represent the fastest progressing segment regardless of the localization in the carotid tree in each individual, the plaques_bulb-prog_, and the plaques_no-bulb-prog_ as well as the progression of ICCAD (ICCAD_mean_ change over time). Validation and precision of the ultrasonographic measurements have been previously reported.^12^

C-IMT_mean_ (mm) in PIVUS was measured at the far wall of the common carotid artery 1–2 cm proximal to the bifurcation. Plaque was defined as c-IMT ≥ 1.5 mm. Plaque area (mm^2^) was measured, and the presence of carotid plaque was annotated.^15,17^

### 2.4 Exploratory analysis in a publicly available dataset

Measures on coronary atherosclerosis were not available in either the IMPROVE or the PIVUS study. For exploratory analyses, we used publicly available data from 208 study participants enrolled in 2012 in the Prospective Comparison of Cardiac PET/CT, SPECT/CT Perfusion Imaging and CT Coronary Angiography With Invasive Coronary Angiography (PACIFIC).^19^ Study participants were >40 years, had a history of new onset of chest pain and intermediate risk for CHD. They underwent a coronary computed tomography angiography (CCTA), and plasma biomarkers were measured by Olink CVD panels II and III. After the exclusion of patients with missing values, 191 were included in the analysis. The absence of CHD was defined as a coronary calcium score of zero and the absence of coronary plaque at CCTA. High-risk-risk plaque was defined as the presence of a coronary lesion with more than two adverse plaque characteristics.^20^

### 2.5 Endotypes identification

Our analytical pipeline is fully described in the Supplementary Material, Methods section ‘Endotype generation and variables selection’^21–28^ and illustrated in Supplementary material online, *Figures S2* and *S3*. Supplementary material online, *Figure S2* (left panel) provides an overview of the analytical pipeline. Briefly, the 124 variables included in the analysis were processed using a multilayer perceptron with an encoder (Encoder-MLP) for dimensional data reduction and c-IMT_mean-max_-related features representation learning as specified in Supplementary material online, *Figure S3*.^21^ This step ensures that features unrelated to c-IMT_mean-max_ are filtered out while those related to c-IMT_mean-max_ are kept in the analysis during the processing of the data. All the continuous variables were standardized (*Z*-standardization) using the formula: (individual value − mean) / (standard deviation). The value of each variable was defined low if below the standardized mean and high if above. For each endotype, we ranked the most relevant variables in the stacking model according to the global |SHAP| value^25,29^ (see Supplementary material online, *Figure S2*, middle panel and right bottom panel). We used the weighted (gene) co-expression network analysis (WGCNA) to identify modules including co-expressed proteins^27,30^ (see Supplementary material online, *Figure S2*, right upper panel) as an attempt to generalize our findings as proteins belonging to the same module might have similar biological functions and thereby be used interchangeably across analytical platforms. Modules were visualized by a dendrogram. Correspondingly, heatmaps were generated for comparison of *Z*-standardized Olink CVD-I protein levels across the four endotypes.

Hereby, endotypes defined by the Encoder-MLP and hierarchical clustering are defined as original endotypes while endotypes replicated by stacking model are defined predicted endotypes.

Python 3.7 was utilized for endotype generation, prediction model building, and model interpretation. The following modules/frameworks in Python were utilized for model development: scikit-learn,^31^ torch,^32^ Optuna,^26^ and SHAP.^25^

### 2.6 Internal and external validation

We built an ensemble stacking model to predict the endotypes and test their replicability. Details are presented in the Supplementary Material ‘Building of the stacking model’ section.

### 2.7 Ethical statement

The IMPROVE study was funded by the Vth European Union (EU) programme and involves seven recruiting centres in five European countries: Finland, France, Italy, the Netherlands, and Sweden. The study was carried out in accordance with the Helsinki Declaration and approved by the IRB at each one of the seven recruiting centres: (i) the Regional Ethics Review Board at Karolinska Institutet, Stockholm, Sweden, (ii) Institutional Review Board (IRB) at the Groupe Hôpitalier Pitie-Salpetriere, Paris, France, (iii) the IRB Comitato Etico delle Aziende Sanitarie della regione Umbria, Perugia and (iv) the IRB at the Ospedale Niguarda Ca´Granda, Milano, both in Italy, (v) the IRB at the University Hospital Groningen, Groningen, the Netherlands, and (vi) the IRB Hospital District of Northern Savo and (vii) the IRB at University of Eastern Finland, both in Kuopio, Finland. Each study participant provided a signed informed consent. The study presented in the present paper was conducted at Karolinska Institutet, Stockholm, Sweden and approved by the Regional Ethical Review Board in Stockholm (2017/404-32).

The PIVUS study was approved by the Ethics Committee of Uppsala University.

### 2.8 Statistical analysis

Continuous variables were reported as mean and standard deviation (Sd) or the median and inter-quartile range if the *P* value of the Shapiro test was <0.05 while discrete variables as numbers and percentages. Violin plots were plotted to compare baseline and progression ultrasonographic carotid measures across the four endotypes. Kaplan–Meier curves were used to describe the ASCVD survival. In the IMPROVE, linear regression models were used to estimate the association of endotypes with c-IMT_mean_, c-IMT_max_, and c-IMT_mean-max_, plaque area, number of plaques, ICCAD at baseline, and with the respective progression measures after 30 months of follow-up of c-IMT_mean-prog_, c-IMT_mean-max-progr_, fastest c-IMT_max-progr_, plaques_bulb-prog_, plaques_no-bulb-prog_ as well as the progression of ICCAD. The fastest c-IMT_max-progr_ was log transformed log10 (fastest c-IMT_max-progr_ + 0.1). Estimates are expressed as coefficients (*β*) and standard error (SE), and endotype 1 was the reference group.

A Cox proportional hazard model was used to estimate the association of the original endotypes 2–4 with the risk of ASCVD in the IMPROVE as compared to endotype 1. Estimates are expressed as hazard ratio (HR) and 95% confidence interval (95% CI). Testing of Schoenfeld residuals confirmed the proportionality of hazards.

Risk estimates from the linear and Cox regression models were adjusted by potential confounders. In IMPROVE in Model 1, we adjusted by latitude, the main c-IMT predictor in the IMPROVE,^14^ and analytical batch used to measure the Olink biomarkers; in Model 2, we included Model 1 + smoking, body mass index, LDL-cholesterol, and diabetes; and in Model 3, we further adjusted by anti-hypertensive, anti-platelet, and lipid-lowering treatment. In Model 2, the corresponding baseline ultrasound measure was included in the model when the outcome was a progression measure, while in the ASCVD risk analysis, we included c-IMT_mean-max_ at baseline. For detecting potential collinearity and evaluate its influence, we estimated variance inflation factors (VIFs). We did not adjust by age, biological sex, and systolic blood pressure (SBP) as these three variables are present in all the four endotypes. Since carotid ultrasound measures were highly correlated,^10,12^ no multiple correction test was performed.

Results were validated internally in the IMPROVE replicated dataset (*n* = 219) using the same outcome measures outlined above for the derived dataset. In addition, we validated our results in PIVUS (*n* = 960). In PIVUS, we estimated the association of the predicted endotypes with c-IMT_mean_, presence of carotid plaque and carotid plaque area, and with the risk of MI and ischaemic stroke. We used the same analytical strategy as described above for the IMPROVE, with the exception of the inclusion of latitude in the regression model.

Finally, we looked at the distribution high-risk coronary artery plaques and prevalence of CHD in the PACIFIC dataset (*n* = 191). Because of very limited number of patients in each predicted endotype group, we did not perform an inference analysis.

SCORE2 and SCORE2-OP are 10-year-CVD risk prediction scores designed for the European population (https://www.escardio.org/Education/Practice-Tools/CVD-prevention-toolbox/SCORE-Risk-Charts).^33,34^ We estimated the *C*-statistics for endotypes, SCORE2, c-IMT_mean-max_, and presence of plaque towards 3-year ASCVD risk and the discrimination improvement by adding endotype to SCORE2, c-IMT_mean-max_, and presence of plaque in IMPROVE cohort. Additionally, we estimated the total net reclassification improvement (NRI) of endotypes as compared to SCORE2 [cut-off thresholds (%) of low (<5), moderate (5–<10), and high (≥10) risks], to the measurement of c-IMT_mean-max_ (as a continuous variable) and to the presence of carotid plaque. In all analyses, we have taken both the NRI due to the re-correct classification of event cases (NRI+) and the NRI due to correct reclassification of non-event cases (NRI−).^35^ Using the original risk classification estimated by the SCORE2 to stratify CV risk in the IMPROVE resulted in an inflated predictive effect of the endotypes (data not shown), probably because of the shortest follow-up time (3 years) and the high-risk profile of study participants in the IMPROVE. Therefore, we built a prediction model using the region-calibrated linear predictors according to the original formula reported in SCORE2.^34^ In PIVUS, we used the SCORE2-OP suggested thresholds (%) of low (<7.5%), moderate (7.5–<15%), and high (≥15%) for 10-year ASCVD risk. For consistency, we also estimated the predictive effect of endotypes in PIVUS using the region-calibrated predictors with the formula reported in SCORE-OP.^33^ The 95% CIs of NRI were estimated based on 1000-time bootstrapping resampling.

R 4.1 was used for the analysis including statistical description and inference. Additional package ‘nricens’^36^ were used for risk stratification improvement evaluation. Two-sided tests were performed for all statistical inferences, and the significant levels in this study were set as 0.05.

## 3. Results

### 3.1 Endotype generation

Hierarchical clustering resulted in the definition of four subclinical carotid atherosclerosis endotypes (see Supplementary material online, *Figures S4* and *S5*). In Supplementary material online, *Figure S5*, the upper left and right panels show the UMAP transformation of 124 variables before and in the middle of Encoder-MLP data processing, while the two bottom panels show the final steps of the extraction of carotid atherosclerosis-related features whereby hierarchical clustering identified four endotypes.^37^ As clusters were modelled on c-IMT_mean-max_, we refer to these clusters as four subclinical carotid atherosclerosis endotypes. We tested this analytical pipeline using as outcome measure random number generator. As shown in Supplementary material online, *Figure S6*, the analysis did not result in any defined cluster. Supplementary material online, *Figure S7* shows the performance of the stacking model in predicting endotypes for PIVUS and PACIFIC.

### 3.2 Endotype characteristics

**Figure 1:**
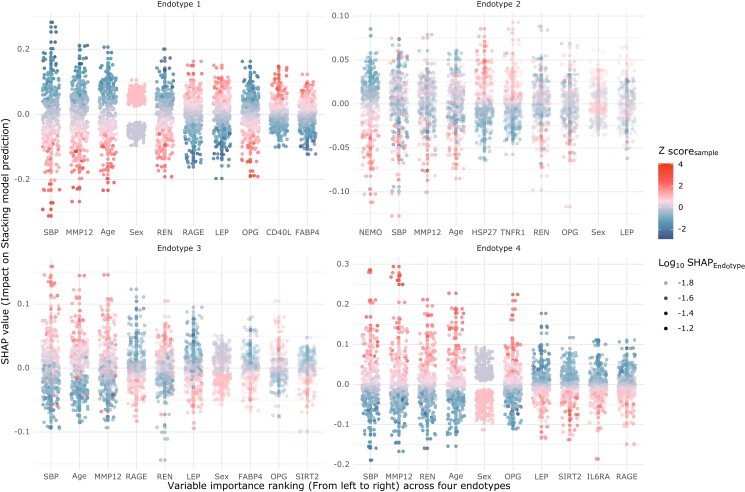
Scatter plot showing the top 10 endotype-specific variables and their impact on endotype prediction. Z-score_sample_ is the standardized value (scaling to mean of 0 and standard deviation of 1) for each variable. The mean of each variable (Z-score_sample_) equals to zero. Z-score_sample_ lower than zero are represented in light blue and Z-score_sample_ over the zero in red. The higher the *Z* score (red dots), the higher the value of original variable. SHAP value: SHapley Additive exPlanations value. It measures the predicted contribution of a variable to one of the four endotypes for each individual. SHAP value of 0 means that a variable does not contribute to predict the endotype. A positive SHAP value indicates that a variable is very likely a predictor of the endotype, while a negative SHAP value indicates in that individual, the variable is not a predictor of the endotype. SHAP_endotype_: SHAP_endotype_ is the mean of |SHAP value| (absolute value). It measures the overall contribution for prediction for a variable to one of the four endotypes (reported with different colours in the Supplementary material online, *Figure S8*). The higher the log10 SHAP_endotype_, the higher the likelihood that the prediction model is accurate. In general, the stacking model predicts endotypes 1 and 4 more reliably as compared to endotypes 2 and 3. In each subplot of endotype, the variables’ importance is ranked from left to right. We randomly sampled 300 individuals in the derived dataset to estimate their SHAP value.

The four endotypes are characterized by shared and specific variables. According to global SHAP value (see Supplementary material online, *Figure S8*), the top five common variables represented in the four endotypes are age, biological sex, SBP, MMP12, and renin. *Figure 1* displays the ranking of variables in each one of the four endotypes based on endotype-specific SHAP value. Age, SBP, and MMP12 although present in all endotypes have opposite effects: young females with low SBP and MMP12 circulating levels were less likely to be assigned to endotypes 3 and 4 as compared to endotypes 1 and 2.

**Figure 2:**
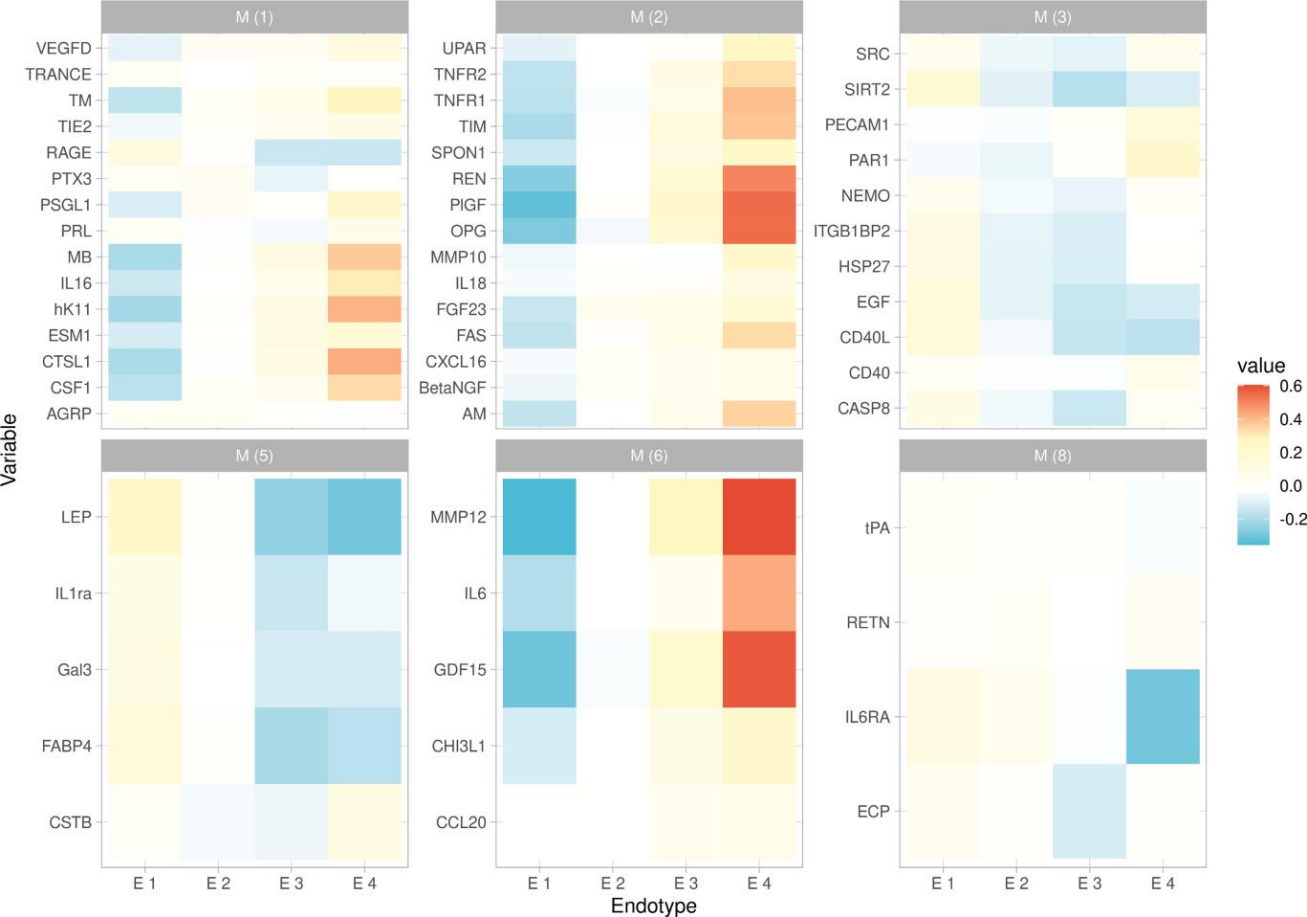
Relative concentrations of biomarkers assigned to endotypes. Biomarkers are grouped into modules of co-expressed protein estimated by the weighted co-expression network analysis to define both protein co-expression modules and relative concentration of each biomarker in the four endotypes. Circulating levels of biomarkers were Z-standardized. Light blue represents the values below the standardized mean, white represents the standardized mean, and red represents the values above standardized mean.

Each endotype shows also specific features. Endotype 1 is characterized by low renin and MMP12 but high level of RAGE and leptin; endotype 2 is characterized by low NEMO and high HSP27 circulating levels; endotype 3 is characterized by low circulating levels of RAGE and leptin, but high renin levels, while endotype 4 was the only one also predicted by the soluble Interleukin 6 receptor, and by high level of OPG. WCGNA identified 17 modules of co-expressed proteins (see Supplementary material online, *Figure S9*). *Figure 2* shows the modules of co-expressed proteins found in the four endotypes as well as the elative circulating levels of each biomarker. Only proteins in modules 1–3, 5, 6, and 8 were represented in the endotypes.

**Figure 3:**
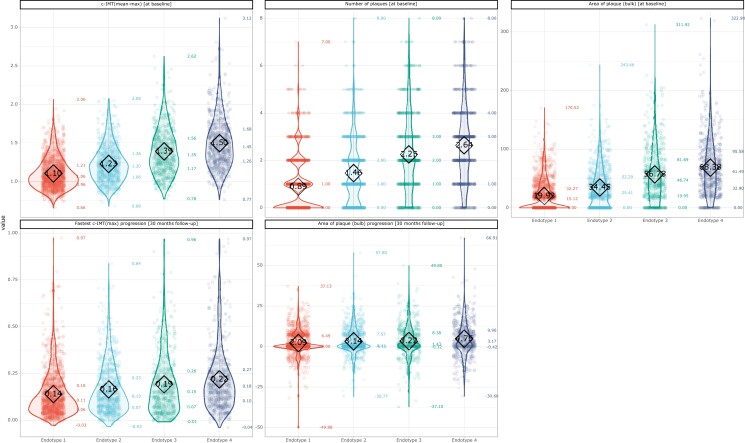
Distribution of ultrasonographic measures across the four endotypes in the derived dataset. Mean value is reported inside a square for each violin plot, while the minimal, 25% quantile, median, 75% quantile, and maximal values are presented at the right side of each violin plot. Violin plots: top-left panel shows the distribution among the four endotypes of the c-IMT_mean-max_ at baseline; top-middle panel shows the distribution among the four endotypes of the number of plaques at baseline; top-right panel shows the distribution the four endotypes of the area of plaque (including bulb) at baseline; the bottom-right panel shows the c-IMT_fastest-progr_ after 30 months follow-up; and the bottom-middle panel shows the area of plaque (including bulb) progression.

As shown in *Table 1* and *Figure 3*, endotype 1 has the most favourable atherosclerotic risk profile. C-IMT measures and plaque area at baseline were progressively thicker and larger, respectively, and progression measures showed a faster rate in endotypes 2, 3, and 4. Consistently, the proportion of ASCVD increased from endotype 1 to endotype 4. ICCAD, which mirrors vascular remodelling more than atherosclerosis, differed between endotypes, but a clear pattern could not be observed, and there were no differences in the ICCAD progression measure across endotypes (*Table 1*). The distribution of all the variables (*n* = 124) included in the analysis across the four endotypes is reported in Supplementary material online, *Table S2*.

**Table 1:** Subclinical carotid atherosclerosis ultrasonographic measures at baseline and after 30 months follow-up (progression) and proportion of ASCVD across the four endotypes in IMPROVE

IMPROVE	Endotype 1	Endotype 2	Endotype 3	Endotype 4	Overall
	(*n* = 1277)	(*n* = 783)	(*n* = 502)	(*n* = 559)	(*n* = 3121)
*Ultrasonographic measures*					
Baseline (mm)					
c-IMT_mean-max_	1.10 (±0.20)	1.23 (±0.24)	1.39 (±0.30)	1.50 (±0.33)	1.25 (±0.30)
c-IMT_mean_	0.79 (±0.14)	0.87 (±0.16)	0.98 (±0.21)	1.05 (±0.22)	0.89 (±0.20)
c-IMT_max_	1.70 (±0.63)	1.97 (±0.69)	2.33 (±0.85)	2.60 (±0.87)	2.03 (±0.81)
ICCAD_mean_	7.46 (±0.67)	7.79 (±0.77)	8.13 (±0.86)	8.45 (±0.87)	7.83 (±0.85)
Number of plaques	0.89 (±1.10)	1.46 (±1.37)	2.25 (±1.67)	2.64 (±1.64)	1.56 (±1.54)
Area of plaques_bulb_	19.93 (±26.20)	34.45 (±34.61)	56.78 (±47.95)	68.38 (±49.14)	38.15 (±41.71)
Area of plaques_no-bulb_	7.09 (±15.14)	14.47 (±22.91)	28.42 (±36.61)	34.82 (±37.26)	17.33 (±28.43)
Progression (mm/year)					
c-IMT_max-progr_	0.04 (±0.15)	0.03 (±0.15)	0.05 (±0.17)	0.06 (±0.19)	0.04 (±0.16)
Fastest c-IMT_max-progr_	0.14 (±0.12)	0.16 (±0.13)	0.19 (±0.16)	0.22 (±0.17)	0.17 (±0.14)
c-IMT_mean-max-progr_	0.02 (±0.04)	0.02 (±0.05)	0.03 (±0.06)	0.03 (±0.06)	0.02 (±0.05)
ICCAD_mean_ change over time	0.00 (±0.03)	0.01 (±0.03)	0.00 (±0.03)	0.01 (±0.03)	0.00 (±0.03)
Area of plaques_bulb-progr_	2.09 (±7.01)	3.14 (±8.56)	3.22 (±10.10)	4.75 (±11.00)	2.99 (±8.76)
Area of plaques_no-bulb-progr_	0.84 (±4.52)	1.62 (±6.48)	2.10 (±8.15)	2.88 (±8.83)	1.59 (±6.60)
ASCVD *n* (%)	43 (3.4)	26 (3.3)	43 (8.6)	54 (9.7)	166 (5.3)
Cardiac events	25 (2.0)	19 (2.4)	24 (4.8)	35 (6.3)	103 (3.3)
Cerebrovascular events	16 (1.3)	7 (0.9)	17 (3.4)	16 (2.9)	56 (1.8)
Peripheral events	2 (0.2)	0 (0.0)	2 (0.4)	3 (0.5)	7 (0.2)

Continuous data are expressed as mean (Sd) unless otherwise indicated. For study participants with multiple ASCVD, we only report the first event.

Missing values in IMPROVE: c-IMT_mean_, *n* = 2; c-IMT_max_, *n* = 2; number of plaque, *n* = 3; Area of plaques_bulb_, *n* = 3; Area of plaques_no-bulb_, *n* = 1; c-IMT_max-progr_, *n* = 338; c-IMT_max-progr_, *n* = 336; c-IMT_fastest-progr_, *n* = 334; c-IMT_mean-progr_, *n* = 334; Area of plaques_bulb-progr_, *n* = 354; Area of plaques_no-bulb-progr_, *n* = 351.

### 3.3 Association of endotypes with c-IMT, carotid plaques, and ICCAD at baseline and after 30 months of follow-up

As shown in *Table 2*, as compared to endotype 1, endotypes 2, 3, and 4 were associated with a more severe atherosclerosis profile. Adjustment for the common CV risk factors and treatment marginally modified the risk estimates. ICCAD changes over time were not significantly different across the four endotypes (all with *P* > 0.05). Adjustment by age and sex did not change the risk estimates (data not shown). Very modest collinearity was observed, with the highest VIF attributed to latitude (1.95, 5 df).^38^

**Table 2:** Association between endotypes and atherosclerosis-related outcomes in IMPROVE: carotid ultrasonographic measures and risk of ASCVD

	Model 1		Model 2		Model 3	
*Carotid ultrasonographic measures at baseline*	β (SE)	*P* value	β (SE)	*P* value	β (SE)	*P* value
c-IMT_mean_						
Endotype 2	0.07 (0.008)	<0.0001	-	-	-	-
Endotype 3	0.17 (0.010)	<0.0001	-	-	-	-
Endotype 4	0.24 (0.009)	<0.0001	-	-	-	-
c-IMT_mean-max_						
Endotype 2	0.11 (0.012)	<0.0001	-	-	-	-
Endotype 3	0.26 (0.014)	<0.0001	-	-	-	-
Endotype 4	0.36 (0.014)	<0.0001	-	-	-	-
c-IMT_max_						
Endotype 2	0.24 (0.034)	<0.0001	-	-	-	-
Endotype 3	0.58 (0.041)	<0.0001	-	-	-	-
Endotype 4	0.84 (0.040)	<0.0001	-	-	-	-
Number of plaques						
Endotype 2	0.51 (0.064)	<0.0001	-	-	-	-
Endotype 3	1.26 (0.077)	<0.0001	-	-	-	-
Endotype 4	1.62 (0.075)	<0.0001	-	-	-	-
Area of plaques_bulb_						
Endotype 2	13.11 (1.74)	<0.0001	-	-	-	-
Endotype 3	34.49 (2.07)	<0.0001	-	-	-	-
Endotype 4	45.49 (2.03)	<0.0001	-	-	-	-
Area of plaques_no-bulb_						
Endotype 2	6.87 (1.23)	<0.0001	-	-	-	-
Endotype 3	20.4 (1.46)	<0.0001	-	-	-	-
Endotype 4	26.86 (1.44)	<0.0001	-	-	-	-
ICCAD_mean_						
Endotype 2	0.30 (0.04)	<0.0001	-	-	-	-
Endotype 3	0.61 (0.04)	<0.0001	-	-	-	-
Endotype 4	0.89 (0.04)	<0.0001	-	-	-	-
*Carotid ultrasonographic progression measures*						
c-IMT_mean-max-progr_						
Endotype 2	0.001 (0.003)	0.74	0.003 (0.003)	0.19	0.003 (0.003)	0.18
Endotype 3	0.004 (0.003)	0.16	0.010 (0.003)	0.001	0.011 (0.003)	0.001
Endotype 4	0.006 (0.003)	0.031	0.015 (0.003)	<0.0001	0.015 (0.003)	<0.0001
Fastest c-IMT_max-progr_						
Endotype 2	0.036 (0.010)	<0.0001	0.026 (0.010)	0.009	0.025 (0.010)	0.012
Endotype 3	0.063 (0.010)	<0.0001	0.036 (0.012)	0.003	0.036 (0.012)	0.002
Endotype 4	0.10 (0.010)	<0.0001	0.061 (0.012)	<0.0001	0.060 (0.013)	<0.0001
Area of plaques_bulb-progr_						
Endotype 2	0.96 (0.43)	0.02	1.37 (0.43)	0.001	1.37 (0.43)	0.001
Endotype 3	0.95 (0.51)	0.06	2.17 (0.53)	<0.0001	2.14 (0.53)	<0.0001
Endotype 4	2.38 (0.51)	<0.0001	4.00 (0.55)	<0.0001	4.00 (0.55)	<0.0001
Area of plaques_no-bulb-progr_						
Endotype 2	0.74 (0.32)	0.023	0.96 (0.32)	0.003	0.97 (0.32)	0.003
Endotype 3	1.19 (0.38)	0.002	1.97 (0.39)	<0.0001	1.96 (0.40)	<0.0001
Endotype 4	1.95 (0.38)	<0.0001	2.95 (0.40)	<0.0001	2.97 (0.41)	<0.0001
ICCAD_mean_ change over time						
Endotype 2	0.002 (0.002)	0.18	0.003 (0.002)	0.07	0.003 (0.002)	0.07
Endotype 3	0.001 (0.002)	0.72	0.002 (0.002)	0.23	0.002 (0.002)	0.24
Endotype 4	0.001 (0.002)	0.59	0.003 (0.002)	0.08	0.003 (0.002)	0.09
ASCVD	HR (95% CI)	*P* value	HR (95% CI)	*P* value	HR (95% CI)	*P* value
Endotype 2	0.97 (0.59–1.62)	0.93	0.89 (0.54–1.48)	0.66	0.87 (0.53–1.46)	0.61
Endotype 3	2.43 (1.53–3.85)	<0.0001	1.91 (1.17–3.10)	0.009	1.96 (1.21–3.12)	0.006
Endotype 4	2.85 (1.82–4.47)	<0.0001	2.02 (1.23–3.32)	0.006	1.96 (1.18–3.23)	0.009
Cardiac events						
Endotype 2	1.28 (0.70–2.36)	0.41	1.21 (0.66–2.22)	0.55	1.25 (0.67–2.30)	0.48
Endotype 3	2.48 (1.37–4.48)	0.003	2.06 (1.11–3.80)	0.022	2.23 (1.20–4.16)	0.011
Endotype 4	3.02 (1.72–5.29)	<0.0001	2.28 (1.22–4.26)	0.01	2.26 (1.20–4.28)	0.012
Cerebrovascular events						
Endotype 2	0.73 (0.29–1.83)	0.50	0.63 (0.25–1.58)	0.32	0.59 (0.24–1.52)	0.28
Endotype 3	2.86 (1.34–6.05)	0.006	2.04 (0.91–4.56)	0.08	2.09 (0.94–4.67)	0.07
Endotype 4	2.85 (1.33 6.08)	0.007	1.78 (0.76–4.16)	0.18	1.71 (0.72–4.03)	0.22

Results of the linear regression analysis are reported as *β* coefficient (standard error), and results of the Cox regression model are reported as HR and (95% CI). Model 1: adjusted Olink analytical batch, latitude. Model 2: Model 1 + diabetes mellitus, LDL-cholesterol, smoking, body mass index, and ultrasonographic measures at baseline; for the regression for log10 (c-IMT_fastest-progr_ + 0.1) adjusted ultrasound measure of c-IMT_max_, and also the regression for ICCAD_mean_progr_ adjusted ICCAD_mean_. The other model adjusted c-IMT_mean-max_. Model 3: Model 2 + anti-platelet treatment, anti-hypertension treatment, and lipid-lowering treatment.

### 3.4 Endotypes and the risk of future ASCVD

**Figure 4:**
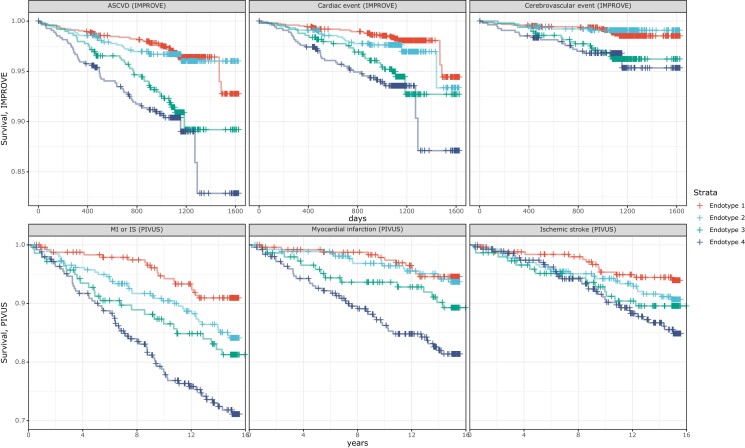
Atherosclerosis-related cardiovascular disease and cardiac events survival curves. The subplots in the upper panel display ASCVD (left), cardiac event (middle), and cerebrovascular event (right) in IMPROVE. The subplots in the bottom panel display myocardial infarction (MI) or ischaemic stroke (IS) (left), myocardial infarction (middle), and ischaemic stroke (right) in PIVUS. Red, endotype 1; light blue, endotype 2; green, endotype 3; dark blue, endotype 4.

As shown in *Figure 4*, endotype 4 showed the lowest ASCVD event-free survival rate followed by endotype 3, while endotype 2 and endotype 1 had a similar event-free survival rate. A similar pattern could be observed when only cardiac and cerebrovascular events were analysed. Consistently, ASCVD risk was significantly higher in endotypes 3 and 4 as compared to endotype 1, with a multi-adjusted HR of 1.96 (95% CI) 1.21–3.19 and HR of 1.96 (95% CI) 1.18–3.23, respectively (see Supplementary material online, *Table S2*). Adjustment by age and sex did not change the risk estimates (data not shown).

### 3.5 Effect of endotypes on measures of discrimination and reclassification

The addition of endotypes corresponded to a modest but consistent increase in *C*-statistics. As compared to SCORE2 (0.62) and endotypes (0.63) alone, the addition of endotypes to the SCORE2 resulted in a *C*-statistic of 0.65; similarly, addition of endotypes to c-IMT_mean-max_ (0.64) resulted in a *C*-statistic of 0.67 and addition of endotypes to the model including presence of plaque (0.58) resulted in a *C*-statistic of 0.66.

As shown in Supplementary material online, *Table S3*, endotypes improved the 3-year ASCVD risk classification predicted by SCORE2, with a global NRI (95% CI) of 0.15 (0.03-0.30). Improvement derives only from event NRI of 0.13 (0.001-0.31), with a non-event global NRI of 0.02 (−0.07-0.11). When c-IMT_mean-max_ was used as a predictor, endotypes improved the prediction model with a global NRI of 0.12 (0.008-0.33) while presence of a carotid plaque was used as a predictor the global NRI improved with a value of 0.32 (0.05–0.55) as shown in Supplementary material online, *Tables S4* and *S5*.

### 3.6 Internal validation

Ultrasonographic measures and proportion of ASCVD showed a similar distribution across the original endotypes and the endotypes predicted by the stacking model (predicted endotypes) in the derived (*n* = 3121) and replicated (*n* = 219) datasets (*Table 1* and Supplementary material online, *Tables S6* and *S7*). Of note, the regression coefficients estimated for the predicted endotypes in the derived dataset were comparable to those observed for the original endotypes (see Supplementary material online, *Table S8* and *Table 2*). As shown in Supplementary material online, *Table S9*, the predicted endotype could also improve the 3-year ASCVD risk reclassification, with a global NRI of 0.17 (0.04–0.31), which is also largely dependent from the analysis of event NRI. We did not perform statistical inference in the replicated dataset due to the limited number of study participants in each predicted endotype.

### 3.7 External validation of endotypes in the PIVUS study

Supplementary material online, *Table S10* shows the distribution of measures of subclinical carotid atherosclerosis, MI, and ischaemic stroke across the four predicted endotypes in PIVUS. As compared to endotype 1, study participants with endotypes 3 and 4 showed a thicker c-IMT and a larger plaque area. As shown in *Table 3*, during 15-year follow-up, the risk for the composite events of MI and ischaemic stroke was higher in endotype 3 with a HR of 2.46 and 95% CI (1.30–4.67) and endotype 4 with a HR of 3.25 and 95% CI (1.83–5.75) as compared to endotype 1. We estimated the NRI for the combined MI and ischaemic stroke as compared to SCORE2-OP. NRI increment within 5 years was due to event NRI as observed in the IMPROVE (3-year ASCVD risk), while at 10-year follow-up, NRI increment mainly resulted from both event and non-event NRI (NRI−) (see Supplementary material online, *Tables S11* and *S12*).

**Table 3:** Association between predicted endotypes and atherosclerosis-related outcomes in PIVUS: carotid ultrasonographic measures and risk of MI and ischaemic stroke

	Model 1		Model 2		Model 3	
*Carotid ultrasonographic measures*	β (SE)	*P* value	β (SE)	*P* value	β (SE)	*P* value
c-IMT_mean_						
Pred endotype 2	0.007 (0.015)	0.65	-	-	-	-
Pred endotype 3	0.045 (0.017)	0.0082	-	-	-	-
Pred endotype 4	0.075 (0.014)	<0.0001	-	-	-	-
Area of plaque						
Pred endotype 2	2.36 (1.49)	0.11	-	-	-	-
Pred endotype 3	6.09 (1.74)	0.0004	-	-	-	-
Pred endotype 4	11.73 (1.47)	<0.0001	-	-	-	-
Presence of carotid plaque	OR (95% CI)	*P* value	OR (95% CI)	*P* value	OR (95% CI)	*P* value
Pred endotype 2	1.41 (0.99–2.03)	0.057	-	-	-	-
Pred endotype 3	2.11 (1.37–3.27)	0.0007	-	-	-	-
Pred endotype 4	4.33 (2.90–6.47)	<0.0001	-	-	-	-
MI or ischaemic stroke	HR (95% CI)	*P* value	HR (95% CI)	*P* value	HR (95% CI)	*P* value
Pred endotype 2	1.82 (1.06–3.13)	0.03	1.89 (1.05–3.42)	0.030	1.89 (1.04–3.42)	0.036
Pred endotype 3	2.26 (1.25–4.08)	0.0071	2.45 (1.30–4.64)	0.006	2.46 (1.30–4.67)	0.005
Pred endotype 4	3.68 (2.22–6.09)	<0.0001	3.29 (1.87–5.79)	<0.0001	3.25 (1.83–5.75)	<0.0001
Myocardial infarction						
Pred endotype 2	1.16 (0.54–1.48)	0.69	1.01 (0.43–2.39)	0.98	1.03 (0.43–2.45)	0.94
Pred endotype 3	1.98 (0.90–4.34)	0.088	1.93 (0.87–4.52)	0.13	1.93 (0.82–4.54)	0.13
Pred endotype 4	3.86 (2.03–4.35)	<0.0001	3.17 (1.52–6.61)	0.002	3.19 (1.52–6.69)	0.002
Ischaemic stroke						
Pred endotype 2	1.99 (0.97–4.08)	0.061	2.47 (1.12–5.43)	0.020	2.42 (1.10–5.34)	0.029
Pred endotype 3	2.00 (0.88–4.55)	0.095	2.57 (1.07–6.19)	0.030	2.62 (1.09–6.34)	0.032
Pred endotype 4	2.91 (1.45–5.83)	0.003	3.02 (1.38–6.62)	0.006	2.98 (1.35–6.59)	0.007

Results of the linear regression analysis are reported as *β* coefficient (standard error), and results of the Cox regression model are reported as HR and 95% CI. Model 1: univariable analysis. Model 2: Model 1 + diabetes mellitus, LDL-cholesterol, smoking, body mass index, and c-IMT at baseline. Model 3: Model 2 + anti-platelet treatment + anti-hypertension treatment + lipid-lowering treatment.

### 3.8 Descriptive analyses in PACIFIC

Patients with predicted endotype 4 in PACIFIC had a higher prevalence of high-risk plaques while the absence of CHD was more common in endotypes 1 and 2 (see Supplementary material online, *Table S13*).

## 4. Discussion

The main finding of our study is the identification of four carotid subclinical atherosclerosis endotypes. Each endotype is characterized by a clinical and molecular signature and mirrors a selective atherosclerotic risk profile. The four endotypes were able, to a certain extent, to improve ASCVD risk prediction.

We have applied a modern analytical approach that combines feature engineering with traditional statistical and epidemiological analyses to extract relevant clinical and biological markers of atherosclerosis. Previous studies^4,39,40^ have used a machine learning approach to disentangle the heterogeneity of cardiovascular or other non-communicable diseases in patient-based population studies, thus defining disease-related endotypes. In the present study, we identified endotypes able to discriminate atherosclerosis profiles before the onset of ASCVD. In this perspective, our results have the potential to contribute to the ongoing efforts to improve ASCVD primary prevention precision medicine-based strategies.^41–43^

We used carotid intima-media thickness to extract features related to atherosclerosis from multi-dimensional data. By doing this, we have defined clusters of individuals defined by endotypes. Then, we used endotypes to stratify the progression of carotid atherosclerosis and risk of ASCVD. Although the endotypes result from 124 variables and c-IMT-related information, the model does not presume the association of the endotype on c-IMT measures. This is reflected by our simulation experiment that failed to force Encoder-MLP to learn independent random number information from the same set of variables, while using c-IMT_mean-max_ to extract atherosclerosis features resulted in four relevant endotypes. During regression analysis, we adjusted for the corresponding baseline ultrasonographic measure to rule out the possibility that proposed endotypes are a merely substitute of c-IMT.

Age, biological sex, and SBP are among the clinical characteristics represented in all endotypes. SBP is an important determinant of c-IMT when atherosclerotic plaques are not included in the measurement. In the IMPROVE, plaques were included in the c-IMT measurement at multiple sites, including the carotid bifurcation and internal carotid artery where the plaque forms and progresses.^44^ While we cannot rule out that the endotypes we have identified also reflect the effect of SBP on the carotid vascular wall, the observation that endotypes associate with c-IMT and with the presence of atherosclerotic plaques and their progression suggests that they mirror the atherosclerotic process. Of note, a clear pattern of association was not observed for ICCAD changes over time, a measure that reflects vascular remodelling more than atherosclerosis.^12^

The four endotypes display common clinical characteristics but are also characterized by unique molecular signatures. Endotypes 1 and 2 show a mild carotid atherosclerosis profile. Endotype 1 individuals were more likely young females, with low SBP, and low circulating renin and MMP12 levels. Endotype 2 revealed a unique molecular signature with relatively low circulating levels of NEMO and MMP12 and high circulating levels of HSP27, yet an atherosclerotic risk profile minimally different from endotype 1. Both NEMO and HSP27 assigned to module 3 by WGCNA participate to molecular pathways able to attenuate atherosclerotic progression.^6^

On the contrary, both endotypes 3 and 4 are associated with an unfavourable atherosclerotic risk profile. Endotype 3 is characterized by high SBP, MMP12, and renin and low RAGE levels. Finally, endotype 4 showed the worst atherosclerotic risk profile. In addition to older age and male sex, high circulating OPG levels characterize this endotype. OPG associates with acute coronary syndromes and CVD-related mortality^45,46^ together with high levels of GDF15,^47,48^ MMP12,^15^ and CHI3L1,^49^ all co-expressed in module 6 by WCGNA.^48,50^ Of note, endotype 4 is characterized by low soluble Interleukin 6 receptor circulating levels, a known pro-inflammatory biomarker.^51^ Analysis by WCGNA identified 6 modules of co-expressed proteins including biomarkers whose relative concentration differed across endotypes.

Progression and regression of subclinical atherosclerosis provide an important window for ASCVD treatment and prevention,^13^ and recent data indicate that progression of atherosclerosis is better detected in peripheral vascular beds, including the carotids, as compared to the coronary arteries also in individuals classified as at low risk of ASCVD according to the currently available risk scores.^11^ Endotypes defined in our study consistently associated with c-IMT progression and risk of future ASCVD. We performed several internal replication analyses to test the robustness of the endotypes. In the IMPROVE replicated cohort, endotypes predicted from stacking model share a similar atherosclerosis risk profile, as the original endotypes from the derived cohort. In the derived cohort, stacking model predicted endotype kept their effects in almost all the analyses. More importantly, the predicted endotypes associated with measures of carotid atherosclerosis and with the risk of MI and ischaemic stroke in an independent cohort, the PIVUS, thus supporting and extending our findings in the IMPROVE. Further, our exploratory analyses in the PACIFIC dataset prompt us to speculate that atherosclerosis profiles derived from measures of carotid subclinical atherosclerosis may be used also to discriminate the presence and/or severity of coronary atherosclerosis. However, previous studies have not consistently shown an association or a correlation between the presence of subclinical atherosclerosis in the peripheral and coronary arteries, the underlying reason being that atherosclerosis is a systemic disease with a broad spectrum of contributing factors.^52,53^ In this perspective, our approach based on endotypes may provide a more comprehensive classification of the atherosclerotic process by including a large number of factors potentially contributing to the progression of the disease. Data in larger studies where coronary atherosclerosis measures are available are warranted to address this point.

As a secondary aim, we estimated if endotypes improved measures of ASCVD risk discrimination and reclassification. Endotypes incremented the discriminatory ability of c-IMT, carotid plaque, and SCORE2 with a magnitude that reflects the regression coefficients and HRs, as observed in other studies.^51^ To observe larger improvements in the discriminatory ability of a novel predictor, a very high HR (>8) is required.^54^ ASCVD risk reclassification^35,36^ in the IMPROVE and PIVUS improved when endotypes were added to the model as compared to SCORE2 but also to measures of subclinical carotid atherosclerosis. Of interest, the NRI improved in the short-term follow-up in the IMPROVE (3 years) and PIVUS (5 years) mostly by reclassifying individuals with a cardiovascular event, while it improved NRI in those events free in the long-term follow-up in PIVUS. However, SCORE2 was designed to estimate the 10-year ASCVD risk rather than 3-year ASCVD risk. Even though we statistically reweighted the coefficients of SCORE2 to adapt to 3-year and 5-year ASCVD risks, we might still use an inappropriate baseline model as reference. As only about 5% of participants developed the ASCVD within 3 years in the IMPROVE and within 5 years in the PIVUS, the effectiveness of risk prediction using endotype might not be as effective in practice. Therefore, even if we observe an improvement in risk prediction, further studies are warranted to test the validity of endotypes as ASCVD risk predictors. Overall, our data support the use of endotypes in clinical cardiology above and beyond the currently available imaging and prediction risk scores. Our data indicate that even within the same risk stratum defined by either measure of subclinical atherosclerosis or a risk prediction model, endotypes can disclose a biological heterogeneity that affects the risk of future cardiovascular events. Implementation in clinical practice needs, however, both studies in other cohorts with available measures of subclinical atherosclerosis in peripheral and coronary arteries and validation of the subset of biomarkers to be measured.

Our study has several strengths and limitations. The IMPROVE cohort is a well-characterized multi-centre prospective population-based cohort in Europe, designed to identify determinants of c-IMT progression, and we have applied a novel analysis strategy using a pre-established unpublished protocol. Main limitations of our study are (i) the lack of knowledge of the mechanisms underlying endotypes: though the endotypes were identified based on 124 variables including 92 OLINK proteins and other atherothrombotic-related biomarkers, there is still limited evidence of which mechanisms are interactively or independently entailed in the derivation of endotypes; (ii) sensitivity to noise: hierarchical clustering is commonly applied in biomedical studies but is sensitive to noise. Therefore, misclassification/mis-clustering of endotype is expected; (iii) almost all the study participants are Caucasian, and therefore, results cannot be generalized to other ethnic groups; (iv) the bulk of our data is derived from observational studies and is limited to carotid atherosclerosis. However, our preliminary data on the PACIFIC suggest that a similar approach could be taken to predict and assess severity of coronary atherosclerosis; (v) the number of ASCVD events is relatively low in both IMPROVE and PIVUS. Therefore, it is hard to perform analyses stratified by biological sex. As circulating levels of biomarkers^55^ differ between men and women, further studies are warranted to define sex-specific endotypes. Further, (vi) we could not analyse the association of endotypes with features of carotid plaques leading to plaque rupture and (vii) because of the relatively low number of events, we used a composite definition of cardiac and cerebral events using both hard and soft endpoints in the IMPROVE. However, we observed comparable risk estimates when we analysed the association of endotypes with MI and ischaemic stroke in PIVUS.

In conclusion, we have identified four endotypes representative of distinct atherosclerotic characteristics and risk profiles associated with progression of carotid atherosclerosis and risk of future cardiovascular events in two independent populations. Our results support the use of endotypes to implement precision medicine in ASCVD prevention.

## Supplementary material

Supplementary material is available at *Cardiovascular Research* online.

## Authors’ contributions

Author contribution conception of the work (Q.S.C., O.B., L.Z., B.G.), data acquisition (D.B., F.V., E.T., A.G., M.P., A.J.S., S.K., K.S., L.L.), data interpretation (Q.S.C., O.B., L.Z., D.B., F.V., P.E., B.G.), drafting the work (Q.S.C., B.G.), revision for important intellectual content (L.Z., R.J.S., P.E., F.V., D.B., O.B.).

## Supplementary Material

cvad106_Supplementary_Data

## Data Availability

Data are available upon reasonable request to the corresponding author. The code used to generate endotypes is available at https://github.com/QSchenKI/Subclinical_athero_endotype/tree/main/.
