# Supplementary material for: A machine learning based approach to identify carotid subclinical atherosclerosis endotypes
Source: Cardiovasc Res. 2023 Jul 21;119(16):2594–606. doi: 10.1093/cvr/cvad106 (PMC10730242; doi:10.1093/cvr/cvad106)
Supplement: cvad106_Supplementary_Data [file cvad106_supplementary_data.docx]

# **Supplementary Material**

# **A machine learning based approach to identify carotid subclinical atherosclerosis endotypes**

Qiao Sen Chen^1^, Otto Bergman^1^, Louise Ziegler^2^ Damiano Baldassarre ^3,4^, Fabrizio Veglia ^5^, Elena Tremoli ^5^, Rona J Strawbridge ^1,6,7^, Antonio Gallo ^8^, Matteo Pirro^9^, Andries J. Smit ^10^, Sudhir Kurl ^11^, Kai Savonen ^12,13,^ Lars Lind^14^, Per Eriksson^1^, Bruna Gigante^1^, ^15^ on behalf of the IMPROVE study group*

^1^ Division of Cardiovascular Medicine, Department of Medicine Solna, Karolinska Institutet, Stockholm, Sweden; ^2^ Division of Medicine and Department of Clinical Sciences, Danderyd Hospital, Karolinska Institutet, Stockholm, Sweden; ^3^ Department of Medical Biotechnology and Translational Medicine, Università di Milano, Milan, Italy; ^4^ Centro Cardiologico Monzino, IRCCS, Milan, Italy; ^5^ Maria Cecilia Hospital, Cotignola (RA), Italy; ^6^ Institute of Health and Wellbeing, University of Glasgow, Glasgow, UK; ^7^ Health Data Research, UK; ^8^ Sorbonne Université, INSERM UMR1166, Lipidology and cardiovascular prevention Unit, Department of Nutrition, APHP, Hôpital Pitié-Salpètriêre, Paris, France; ^9^ Internal Medicine, Angiology and Arteriosclerosis Diseases, Department of Medicine, University of Perugia, Perugia, Italy; ^10^ Department of Medicine, University Medical Center Groningen, Groningen & Isala Clinics Zwolle, Department of Medicine, the Netherlands; ^11^ Institute of Public Health and Clinical Nutrition, University of Eastern Finland, Kuopio Campus, Finland; ^12^ Kuopio Research Institute of Exercise Medicine, Kuopio, Finland; ^13^ Department of Clinical Physiology and Nuclear Medicine, Science Service Center, Kuopio University Hospital, Kuopio, Finland; ^14^ Department of Medical Sciences Uppsala University Uppsala Sweden; ^15^ Department of Cardiology, Danderyd University Hospital, Stockholm, Sweden

Additional member of the IMPROVE study group

C.R. Sirtori^1^, S. Castelnuovo^1^, M. Amato^2^, B. Frigerio^2^, A. Ravani^2^, D. Sansaro^2^, C. Tedesco^2^, A. Bonomi^2^, F. Laguzzi ^3^, Karin Leander^3^, Angela Silveira ^4^, J. Cooper^5^, J. Acharya^5^, K. Huttunen^6^, E. Rauramaa^6^, H Pekkarinen^6^, I.M. Penttila^6^, J. Törrönen^6^, A.I. van Gessel^7^, A.M van Roon^7^, G.C. Teune^7^, W.D. Kuipers^7^, M. Bruin^7^, A. Nicolai^6^7 P. Haarsma-Jorritsma^7^, D.J. Mulder^7^, H.J.G. Bilo^7^, G.H. Smeets^7^, J.L. Beaudeux^8^, J.F. Kahn^8^, V. Carreau^8^, A. Kontush^8^, J. Karppi^9^, T. Nurmi^9^, K. Nyyssönen^9^, R. Salonen^9^, T.P. Tuomainen^9^, J.Tuomainen^9^, J. Kauhanen^9^, G. Vaudo^10^, A. Alaeddin^10^, D. Siepi^10^, G. Lupattelli^10^, G. Schillaci^10^.

1. Dipartimento di Scienze Farmacologiche e Biomolecolari, Università di Milano, Milan, Italy.
2. Centro Cardiologico Monzino, IRCCS, Milan Italy.
3. Division of Cardiovascular and Nutritional Epidemiology, Institute of Environmental Medicine, Karolinska Institutet, Stockholm, Sweden
4. Division of Cardiovascular Medicine, Department of Medicine Solna, Karolinska Institutet, Stockholm, Sweden
5. University College of London, Department of Medicine, Rayne Institute, London, United Kingdom
6. Foundation for Research in Health Exercise and Nutrition, Kuopio Research Institute of Exercise Medicine, Kuopio, Finland.
7. Department of Medicine, University Medical Center Groningen, Groningen & Isala Clinics Zwolle, Department of Medicine; the Netherlands
8. Assistance Publique - Hopitaux de Paris; Service Endocrinologie-Metabolisme, Groupe Hôpitalier Pitie-Salpetriere, Unités de Prévention Cardiovasculaire, Paris, France.
9. Institute of Public Health and Clinical Nutrition, University of Eastern Finland, Kuopio Campus.
10. Internal Medicine, Angiology and Arteriosclerosis Diseases, Department of Clinical and Experimental Medicine, University of Perugia, Perugia, Italy.

## **Ethical statement**

The IMPROVE study was funded by the Vth European Union (EU) programme and involves seven recruiting centers in five European countries: Finland, France, Italy, the Netherlands, and Sweden. The study was carried out in accordance with the Helsinki Declaration and approved by the IRB at each one of the seven recruiting centers: (1) the Regional Ethics Review Board at Karolinska Institutet, Stockholm Sweden, (2) Institutional Review Board (IRB ) at the Groupe Hôpitalier Pitie-Salpetriere, Paris, France, (3) the IRB Comitato Etico delle Aziende Sanitarie della regione Umbria, Perugia and (4) the IRB at the Ospedale Niguarda Ca´Granda, Milano, both in Italy, (5) the IRB at the University Hospital Groningen, Groningen, the Netherlands, (6) the IRB Hospital District of Northern Savo and (7) and the IRB at University of Eastern Finland, both in Kuopio, Finland. Each study participant provided a signed informed consent. The study presented in the present paper was conducted at Karolinska Institutet, Stockholm, Sweden and approved by the Regional Ethical Review Board in Stockholm (2017/404-32).

The PIVUS study was approved by the Ethics Committee of Uppsala University.

# **Supplementary methods**

## **Definition of the endotype derived variables**

Cardiovascular related demographics, anthropometric, lifestyle, history of disease and biomarkers variables (n=124) from the IMPROVE study *^14^* were included in the present analysis. *Table S1* summarizes the definitions used for the clinical and anthropometric variables, while for biomarkers, it reports the original publication where the method used for measurement is described. Briefly, blood cells count, lipid, glucose and creatinine levels were measured by standard biochemical methods. Body mass index (BMI) was defined as the ratio of the weight to the square of the height (kg/m2) and waist hip ratio (WHR) as the ratio of the circumference of the waist and the hips without normalizing by sex. Renal function was estimated by glomerular filtration rate (eGFR) using the Cockcroft and Gault formula.

## **Endotype generation and variables selection**

### ***Feature engineering***

#### General description

Encoder-MLP is a multilayer perceptron (MLP) with a funnel like architecture, consisting of one encoder and two fully connected layers. The encoder receives the original variables included in the model and transfers them as codes through several hidden layers with sequentially decreased computational units for dimensional data reduction. The codes can be further processed through two fully connected layers to predict the value of output layers*.* The Encoder-MLP can extract linear and non-linear relationship simultaneously, upon activation of specific functions, such as Tanh or Relu.*^21^*

#### Application of Encoder-MLP in present study.

We used Encoder-MLP for feature engineering.*^21^* We input 124 variables (*Table S1*) and adopted Encoder-MLP for data dimensional reduction. C-IMT_mean-max_ was chosen as label to filter the data from the original 124 variables throughout 8 hidden layers. At the end of the computation, the Encoder-MLP extracts codes that can be interpreted as representation (embedding) of c-IMT_mean-max_. We termed the information extracted from the hidden layers of Encoder-MLP as codes-MLP *(Figure S3)*.

We performed bias-variance trade-off experiments to tune the optimal number of the codes by 1000-times Bayesian optimization hyperparameter tuning. We found that 8 codes-MLP had sufficient capacity to reflect the c-IMT_mean-max_ heterogeneity and achieve the best predictive performance (*https://github.com/QSchenKI/Subclinical_athero_endotype/tree/main/log*). These 8 codes-MLP are then used for cluster analysis.

We did a simulation study to analyze the feature extraction process from original 124 variables using an independent random number with the same distribution measures as c-IMT_mean-max_. as outcome measure.

### ***Cluster analysis***

#### Choice of algorithm to define the optimal number of clusters in the IMPROVE

We used hierarchical clustering and further applied NbClust package*^22^* to define the optimal number of clusters. Based on 20 metrics (KL, CH, Hartigan, CCC, Scott, Marriot, TrCovW, TraceW, Friedman, Rubin, Cindex, DB, Silhouette, Ratkowsky, Ball, PtBiserial, McClain, Dunn, SDindex, SDbw) and using a range of number of clusters from 3 to 15, 4 clusters were identified as the optimal number according to the majority rule *(Figure S4*). As c-IMT_mean-max_ was used to filter data, we refer to these 4 clusters as sub-clinical carotid atherosclerosis endotypes based on c-IMT measures. *Figure S5* summarizes the analytical process leading to the definition of the 4 endotypes from the original 124 variables using c-IMT_mean-max_ as outcome variable, while *Figure S6* shows the results of the analysis using the same analytical pipeline but a random number generator with the same distribution c-IMT_mean-max_ as outcome.

### ***Building of the stacking model***

#### General description

Stacking models are a subgroup of ensemble models used to improve the accuracy of classification. Usually, they have a two-layer structure. The first layer contains several base models and the second layer contains an aggregative model to summarize the result from the base model and predict the label of interest. *^23^* The base models usually require diverse and strong classifiers such as logistic regression and support vector machine. After determining the base models, one can stack all the base models together and use another model (commonly a (multinominal) logistic regression) to link the predictive value of each base model.

#### Procedure to build the stacking model

We built an ensemble stacking model to predict the endotypes and test their replicability. We used the four endotypes identified in the cluster analysis as label to train the stacking model. The stacking model consists of the following base models: Extreme Gradient Boosting (XGBoost), logistic regression, and support vector machine.

Three stacking models were constructed. The first one for model interpretation incorporates 99 variables without missing values in participants in the IMPROVE replication dataset (n=219). They included age, sex, SBP, DBP, total cholesterol and glucose levels, CRP, and the proteins from the Olink CVD-I panel. The second one, was designed for PIVUS dataset and incorporates 77 variables. All the traditional CV risk factors were included in this model, and harmonization of Olink CVD-I, II and III panels was performed. *(*[*https://github.com/QSchenKI/Subclinical_athero_endotype/tree/main/*](https://github.com/QSchenKI/Subclinical_athero_endotype/tree/main/)*)*. Finally, a third model was used to predict endotypes in PACIFIC. This model incorporates 91 variables.

We adopted nested cross-validation: first, the derived dataset was split into a training dataset (75%) and a testing dataset (25%). The training dataset was randomly split into 5 folds, 4 (80%) were used as training folds and 1 (20%) as a validation fold. In training folds, the base model was tuned using Bayesian optimization to select the hyperparameter and fit the model using the training fold data and then test the metric of loss function in the validation fold for evaluation. In the validation fold, the hyperparameter of the base model is fixed, but the second layer model starts to train via Bayesian optimization. After tuning all the hyperparameters in both the training and the validation folds, the training dataset was used to fit a stacking model with the hyperparameters list trained by the above-stated procedures. The testing dataset was used only once to evaluate the overall stacking model performance. We used macro-area under the curve (AUC) to evaluate the performance. Each base model individually has to achieve more than 0.7 macro-AUC in the validation fold. Macro-AUC refers to the average of one-versus-rest AUC for each endotype. Cohen’s weighted kappa were used to check the consistency among the three stacking models as shown in *Figure S7.*

### ***Selection of the variables that define each endotype***

#### General description

The SHapley Additive exPlanations (SHAP) compares the difference in model performance difference in any subset of data with and without a specific variable, thereby measuring the marginal contribution of a specific predictor. *^25^*

#### SHAP value for global and endotype specific variable selection

We randomly sampled 300 individuals in the IMPROVE derived dataset and calculated the absolute SHAP value for each one of the 99 variables used in the stacking model using Kernel method. For each endotype, we calculated the endotype specific global SHAP value for each variable. Then we stack all the endotype specific global SHAP values together to get the overall prediction contribution of each specific variable.

### ***Hyperparameter tuning***

Hyperparameters refer to a set of parameters controlling the learning in the machine learning algorithm. Optuna framework was adopted for hyperparameter tuning.*^26^* For hyperparameter tuning of neural network, we used the mean square error as the objective function and tuned 1000 trials to get the optimal hyperparameter list. For stacking model tuning, the objective of the Bayesian optimization to maximize the average of macro-AUC during 5-fold cross validation. Base models have a much more complex hyperparameters space (especially XGboost), so we used 1200 trials (1000 times for shortening the range of hyperparameter space and 200 times for fine tuning). Second layer model is a simple logistic regression with penalty term, and therefore we tune it 100 times only.

### ***Weighted gene co-expression network analysis***

Weighted gene co-expression network analysis (WGCNA) facilitates the identification of co-expressed molecular modules thus facilitating the understanding of possible molecular interactions. *^27,30^* We conducted WGCNA to study co-expressed protein modules in IMPROVE. Firstly, the pair-wise Pearson correlation between Olink CVD-I proteins was estimated. Then we performed the network topology analysis to determine the power threshold for creating the adjacency matrix using the Pearson correlation matrix. This analysis results in the power of 5 for further co-expressed module identification. We set the argument of Deepsplit with 4 and minimal cluster size as 2 during identification of co-expressed modules.

## **Supplementary Tables**

### **Table S1.** List and definition of cardiovascular related demographic, anthropometric, lifestyle, history of disease, and biomarker variables (n=124) used to generate endotypes. For each biomarker, we report the PMID of the original publication where the method used for measurement is described.

| **LIST** | **DEFINITION** |
| --- | --- |
| ***Demographics*** |  |
| Sex | Female/Male |
| Age |  |
| ***Anthropometric variables (abbreviation), (unit of measure)*** | |
| Body Mass Index (BMI), (kg/m2) | |
| Waist-hip ratio (WHR) |  |
| Systolic blood pressure (SBP), (mmHg) | |
| Diastolic blood pressure (DBP), (mmHg) | |
| *Blood* |  |
| Hemoglobin (HB), (g/dL) |  |
| Platelets (Plt) (10^9/L |  |
| Lymphocytes, 10^9/L |  |
| Neutrophils, 10^9/L |  |
| Neutrophils/ Lymphocytes ratio (NLR) | |
| *Metabolic profile* |  |
| Total cholesterol, mmol/L |  |
| LDL cholesterol, mmol/L |  |
| HDL cholesterol, mmol/L |  |
| Triglycerides, mmol/L |  |
| Glucose, mmol/L |  |
| CRP, mmol/L |  |
| eGFR | [140-Age (year)] * Weight (kg) / [72*Creatinine (micromole) *0.0113] (X0.85 in women) |
| **Cardiovascular risk factors** |  |
| Smoking (current/former never) | Smoking currently at least 10 cigarettes/day for at least thirty months |
| Physical activity (Low/Moderate/High) | Brisk walk for ten minutes less than once a week/ two-three times a week/ more than two-three times a week |
| Hypertension (yes/no) | Self-reported and/or diastolic blood pressure (DBP) ≥ 90 mmHg and/or systolic blood pressure (SBP) ≥140 mmHg and/or treatment with anti-hypertensive drugs |
| Diabetes (yes/no) | Self-reported and/or blood glucose level≥ 7 mmol/L and/or treatment with insulin or oral hypoglycaemic drugs. |
| ***Biomarkers (unit)*** | **PMID** |
| IgG antiApoBp210nat | 24401246 |
| IgM antiApoBp210mda | 24401246 |
| Vitamin D (nmol/L) | 24663808 |
| Adiponectin (µg/mL) | 26276317 |
| IgM antiPC (U/mL) | 25150937 |
| IL5 (pg/ml) | 25587992 |
| CD93 (ng/mL) | 27659228 |
| Proinsulin (pmol/L) | 29040868 |
| Insulin (pmol/L) | 29040868 |
| sgp130 (ng/mL) | 31932740 |
| *CVD panel I, Olink* | 28369058 |
| *Biomarker name (abbreviation) All measured in arbitrary units (AU)* | |
| Adrenomedullin (AM) |  |
| Agouti-related protein (AGRP) | |
| Angiopoietin-1 receptor (TIE2) | |
| Beta-nerve growth factor (Beta-NGF) | |
| Caspase-8 (CASP-8) |  |
| Cathepsin D (CTSD) |  |
| Cathepsin L1 (CTSL1) |  |
| C-C motif chemokine 20 (CCL20) | |
| C-C motif chemokine 3 (CCL3) | |
| C-C motif chemokine 4 (CCL4) | |
| CD40 ligand (CD40L) |  |
| Chitinase-3-like protein 1 (CHI3LI) | |
| C-X-C motif chemokine 1 (CXCL1) | |
| C-X-C motif chemokine 16 (CXCL16) | |
| C-X-C motif chemokine 6 (CXCL6) | |
| Cystatin-B (CSTB) |  |
| Dickkopf-related protein 1 (Dkk-1) | |
| Endothelial cell-specific molecule 1 (ESM-1) | |
| Eosinophil cationic protein (ECP) | |
| Epidermal growth factor (EGF) | |
| E-selectin (SELE) |  |
| Fatty acid-binding protein, adipocyte (FABP4) | |
| Fibroblast growth factor 23 (FGF-23) | |
| Follistatin (FS) |  |
| Fractalkine (CX3CL1) |  |
| Galanin peptides (GAL) |  |
| Galectin-3 (Gal-3) |  |
| Growth differentiation factor 15 (GDF-15) | |
| Growth hormone (GH) |  |
| Heat shock 27 kDa protein (HSP 27) | |
| Heparin-binding EGF-like growth factor (HB-EGF) | |
| Hepatocyte growth factor (HGF) | |
| Interleukin-1 receptor antagonist protein (IL-1ra) | |
| Interleukin-16 (IL16) |  |
| Interleukin-18 (IL-18) |  |
| Interleukin-27 subunit alpha (IL27-A) | |
| Interleukin-4 (IL-4) |  |
| Interleukin-6 (IL-6) |  |
| Interleukin-6 receptor subunit alpha (IL-6RA) | |
| Interleukin-8 (IL-8) |  |
| Kallikrein-11 (hK11) |  |
| Kallikrein-6 (KLK6) |  |
| Lectin-like oxidized LDL receptor 1 (LOX-1) | |
| Leptin (LEP) |  |
| Macrophage colony-stimulating factor 1 (CSF-1) | |
| Matrix metalloproteinase-1 (MMP-1) | |
| Matrix metalloproteinase-10 (MMP-10) | |
| Matrix metalloproteinase-12 (MMP-12) | |
| Matrix metalloproteinase-3 (MMP-3) | |
| Matrix metalloproteinase-7 (MMP-7) | |
| Melusin (ITGB1BP2) |  |
| Membrane-bound aminopeptidase P (mAmP) | |
| Monocyte chemotactic protein 1 (MCP-1) | |
| Myeloperoxidase (MPO) |  |
| Myoglobin (MB) |  |
| Natriuretic peptides B (BNP) | |
| NF-kappa-B essential modulator (NEMO) | |
| N-terminal pro-B-type natriuretic peptide (NT-proBNP) | |
| Osteoprotegerin (OPG) |  |
| Ovanrian cancer-related tumor marker CA 125 (CA125) | |
| Pappalysin-1 (PAPPA) |  |
| Pentraxin-related protein PTX3 (PTX3) | |
| Placenta growth factor (PlGF) | |
| Platelet endothelial cell adhesion molecule (PECAM-1) | |
| Platelet-derived growth factor subunit B (PDGF subunit B) | |
| Prolactin (PRL) |  |
| Protein S100-A12 (EN-RAGE) | |
| Proteinase-activated receptor 1 (PAR-1) | |
| Proto-oncogene tyrosine-protein kinase Src (SRC) | |
| P-selectin glycoprotein ligand 1 (PSGL-1) | |
| Receptor for advanced glycosylation end products (RAGE) | |
| Renin (REN) |  |
| Resistin (RETN) |  |
| SIR2-like protein (SIRT2) |  |
| Spondin-1 (SPON1) |  |
| ST2 protein (ST2) |  |
| Stem cell factor (SCF) |  |
| Thrombomodulin (TM) |  |
| TIM-1 (TIM) |  |
| Tissue factor (TF) |  |
| Tissue-type plasminogen activator (t-PA) | |
| TNF-related activation-induced cytokine (TRANCE) | |
| TNF-related apoptosis-inducing ligand (TRAIL) | |
| TNF-related apoptosis-inducing ligand receptor 2 (TRAIL-R2) | |
| Tumour necrosis factor ligand superfamily member 14 (TNFSF14) | |
| Tumour necrosis factor receptor 1 (TNF-R1) | |
| Tumour necrosis factor receptor 2 (TNF-R2) | |
| Tumour necrosis factor receptor superfamily member 5 (CD40) | |
| Tumour necrosis factor receptor superfamily member 6 (FAS) | |
| Urokinase plasminogen activator surface receptor (U-PAR) | |
| Vascular endothelial growth factor A (VEGF-A) | |

### **Table S2.** Distribution of cardiovascular related demographic, anthropometric, lifestyle, history of disease, and biomarker variables (n=124) included in the analysis in the IMPROVE across the 4 endotypes.

|  | **Endotype 1** | **Endotype 2** | **Endotype 3** | **Endotype 4** | **Overall** |
| --- | --- | --- | --- | --- | --- |
|  | **(N=1277)** | **(N=783)** | **(N=502)** | **(N=559)** | **(N=3121)** |
| **Age, yrs** | 61.02 (58.10, 65.64) | 64.81 (59.69, 67.07) | 66.70 (64.08, 68.71) | 67.26 (65.66, 71.46) | 64.67 (59.78, 67.25) |
| **Female/Male n(%)** | 917 (71.8)/ 360 (28.2) | 415 (53.0)/ 368 (47.0) | 177 (35.3)/ 325 (64.7) | 117 (20.9)/ 442 (79.1) | 1626 (52.1)/ 1495 (47.9) |
| **Latitude n(%)** |  |  |  |  |  |
| 43 | 346 (27.1) | 95 (12.1) | 17 (3.4) | 8 (1.4) | 466 (14.9) |
| 45 | 214 (16.8) | 141 (18.0) | 76 (15.1) | 41 (7.3) | 472 (15.1) |
| 48 | 250 (19.6) | 91 (11.6) | 37 (7.4) | 35 (6.3) | 413 (13.2) |
| 53 | 158 (12.4) | 80 (10.2) | 59 (11.8) | 106 (19.0) | 403 (12.9) |
| 59 | 108 (8.5) | 152 (19.4) | 127 (25.3) | 109 (19.5) | 496 (15.9) |
| 62 | 201 (15.7) | 224 (28.6) | 186 (37.1) | 260 (46.5) | 871 (27.9) |
| **Current smoker/ never or former smoker n (%)** | 133 (10.4)/ 1144 (89.6) | 107 (13.7)/ 676 (86.3) | 93 (18.5)/ 409 (81.5) | 122 (21.8)/ 437 (78.2) | 455 (14.6)/ 2666 (85.4) |
| **Physical activity n (%)** |  |  |  |  |  |
| Low/Middle/High | 325 (25)/ 577 (45)/ 375 (29) | 141 (18)/ 366 (47)/ 276 (35) | 75 (15)/ 215 (43)/ 212 (42) | 72 (13)/ 236 (42)/251 (45) | 613 (20)/ 1394 (45)/ 1114 (36) |
| ***Physical examination*** |  |  |  |  |  |
| Body mass index | 26.57 (23.94, 29.40) | 26.81 (24.14, 29.20) | 26.59 (24.58, 29.27) | 27.30 (24.84, 29.71) | 26.78 (24.24, 29.39) |
| Waist-to-hip ratio | 0.90 (0.84, 0.95) | 0.91 (0.85, 0.97) | 0.93 (±0.08) | 0.94 (0.90, 1.00) | 0.92 (0.86, 0.97) |
| Systolic blood pressure, mmHg | 135 (125.00, 147.00) | 141.00 (130.00, 152.00) | 145.00 (135.00, 158.00) | 150.00 (139.00, 162.00) | 140.00 (130.00, 153.00) |
| Diastolic blood pressure, mmHg | 80 (74.00, 88.00) | 82.00 (77.00, 89.50) | 83.00 (78.00, 90.00) | 82.00 (77.00, 90.00) | 82.00 (75.00, 88.00) |
| ***Comorbidities*** |  |  |  |  |  |
| Hypertension n (%) | 878 (68.8) | 638 (81.5) | 450 (89.6) | 515 (92.1) | 2481 (79.5) |
| Diabetes n (%) | 262 (20.5) | 188 (24.0) | 143 (28.5) | 218 (39.0) | 811 (%26.0) |
| ***Blood cells count and hemoglobin*** |  |  |  |  |  |
| Hemoglobin, g/L | 14.00 (13.30, 14.70) | 14.27 (±1.17) | 14.35 (±1.19) | 14.44 (±1.15) | 14.10 (13.40, 15.00) |
| Platelets, 10^9/L | 236.00 (205.00, 273.00) | 232.00 (199.00, 267.00) | 228.50 (197.00, 264.00) | 224.00 (186.50, 262.00) | 232.00 (199.00, 269.00) |
| Lymphocytes, 10^9/L | 33.44 (±8.13) | 32.70 (27.48, 37.90) | 32.73 (±7.95) | 31.40 (26.64, 37.00) | 32.70 (27.36, 38.10) |
| Neutrophils, 10^9/L | 56.90 (±8.82) | 57.50 (52.00, 62.95) | 57.30 (51.80, 63.00) | 58.00 (51.95, 65.00) | 57.30 (51.50, 63.10) |
| NLR | 1.70 (1.32, 2.24) | 1.76 (1.40, 2.26) | 1.77 (1.39, 2.27) | 1.83 (1.42, 2.41) | 1.76 (1.37, 2.29) |
| ***Metabolic profile*** |  |  |  |  |  |
| Glucose, mmol/L | 5.30 (4.80, 5.90) | 5.56 (5.00, 6.30) | 5.70 (5.20, 6.54) | 5.90 (5.30, 7.00) | 5.50 (5.00, 6.30) |
| eGFR ml/min/1,73m^2^ | 81.18 (69.89, 95.19) | 80.91 (66.99, 97.17) | 79.91 (67.24, 96.46) | 82.01 (67.31, 95.68) | 81.09 (68.21, 96.15) |
| Total cholesterol, mmol/L | 5.63 (4.83, 6.42) | 5.47 (±1.11) | 5.23 (4.53, 6.10) | 5.19 (±1.01) | 5.43 (4.69, 6.23) |
| LDL Cholesterol, mmol/L | 3.59 (2.94, 4.32) | 3.54 (2.79, 4.27) | 3.40 (2.80, 4.15) | 3.38 (±0.89) | 3.50 (2.83, 4.21) |
| HDL Cholesterol, mmol/L | 1.30 (1.08, 1.57) | 1.20 (1.02, 1.43) | 1.15 (0.98, 1.39) | 1.10 (0.94, 1.35) | 1.21 (1.02, 1.47) |
| Triglycerides, mmol/L | 1.30 (0.94, 1.88) | 1.29 (0.95, 1.88) | 1.26 (0.90, 1.77) | 1.28 (0.92, 1.82) | 1.29 (0.93, 1.85) |
| ***Biomarkers*** |  |  |  |  |  |
| CRP, mg/L | 2.01 (0.85, 3.79) | 1.75 (0.72, 3.44) | 1.66 (0.60, 3.25) | 1.77 (0.75, 3.45) | 1.83 (0.76, 3.54) |
| IgG antiApoBp210nat | 0.85 (0.68, 1.04) | 0.83 (0.67, 1.04) | 0.83 (0.68, 1.02) | 0.80 (0.64, 1.02) | 0.83 (0.67, 1.04) |
| IgM antiApoBp210mda | 0.99 (0.88, 1.05) | 0.96 (0.81, 1.04) | 0.97 (0.81, 1.05) | 0.96 (0.83, 1.04) | 0.98 (0.85, 1.05) |
| Vitamin D, nmol/L | 46.00 (30.00, 61.00) | 49.00 (35.00, 62.00) | 53.00 (37.00, 69.75) | 52.00 (38.00, 66.00) | 49.00 (34.00, 64.00) |
| Adiponectin, µg/mL | 13.00 (8.18, 20.53) | 11.04 (6.26, 17.83) | 9.16 (5.28, 15.12) | 8.12 (4.98, 12.64) | 10.78 (6.39, 17.63) |
| IgM antiPC, U/mL | 66.00 (41.00, 102.00) | 65.00 (38.50, 103.00) | 60.50 (39.00, 100.50) | 61.00 (36.00, 101.50) | 64.00 (40.00, 102.00) |
| IL5, pg/ml | 0.38 (0.23, 0.66) | 0.42 (0.26, 0.68) | 0.45 (0.30, 0.73) | 0.52 (0.33, 0.77) | 0.43 (0.26, 0.70) |
| CD93, ng/mL | 152.33 (130.24, 180.13) | 155.28 (133.49, 181.23) | 157.73 (136.78, 184.64) | 165.96 (140.42, 190.27) | 156.18 (133.99, 183.83) |
| Proinsulin, pmol/L | 4.92 (3.53, 7.31) | 4.99 (3.35, 8.18) | 5.63 (3.67, 8.41) | 6.22 (4.01, 9.55) | 5.20 (3.64, 8.12) |
| Insulin, pmol/L | 28.58 (16.12, 51.22) | 28.40 (15.75, 50.30) | 26.20 (15.77, 56.45) | 27.71 (16.42, 51.74) | 28.15 (15.99, 51.86) |
| sgp130 | 572.22 (454.68, 706.95) | 552.82 (440.67, 694.57) | 555.81 (444.86, 696.06) | 580.65 (460.04, 728.23) | 566.11 (450.56, 704.69) |
| *Olink CVD I protein, (AU)* |  |  |  |  |  |
| IL8 | 5.19 (4.79, 5.62) | 5.33 (4.93, 5.73) | 5.49 (5.14, 5.89) | 5.66 (5.26, 6.02) | 5.36 (4.93, 5.78) |
| VEGFA | 10.53 (10.29, 10.84) | 10.55 (10.31, 10.83) | 10.55 (10.31, 10.85) | 10.62 (10.39, 10.86) | 10.56 (10.32, 10.84) |
| AM | 7.29 (7.01, 7.56) | 7.34 (±0.45) | 7.38 (±0.46) | 7.50 (7.24, 7.79) | 7.35 (7.06, 7.65) |
| CD40L | 5.98 (5.07, 7.41) | 5.68 (4.92, 6.74) | 5.55 (4.87, 6.45) | 5.52 (4.84, 6.62) | 5.75 (4.94, 6.92) |
| GDF15 | 9.59 (9.28, 9.93) | 9.77 (9.43, 10.12) | 9.90 (9.58, 10.33) | 10.11 (9.77, 10.55) | 9.77 (9.42, 10.19) |
| PlGF | 7.51 (7.31, 7.71) | 7.61 (7.39, 7.86) | 7.67 (7.46, 7.93) | 7.80 (7.59, 8.02) | 7.61 (7.39, 7.85) |
| SELE | 5.85 (±0.66) | 5.82 (±0.70) | 5.86 (5.46, 6.30) | 5.98 (5.53, 6.40) | 5.87 (5.42, 6.32) |
| EGF | 4.75 (3.67, 6.13) | 4.40 (3.51, 5.52) | 4.36 (3.37, 5.27) | 4.36 (3.45, 5.44) | 4.52 (3.55, 5.72) |
| OPG | 9.82 (9.60, 10.03) | 9.92 (±0.37) | 10.00 (9.71, 10.26) | 10.10 (9.89, 10.38) | 9.92 (9.68, 10.17) |
| SRC | 6.20 (4.46, 7.27) | 5.81 (4.39, 7.04) | 5.85 (4.44, 6.90) | 5.91 (4.80, 7.18) | 5.95 (4.49, 7.15) |
| IL1ra | 4.82 (4.42, 5.33) | 4.75 (4.33, 5.26) | 4.68 (4.26, 5.14) | 4.70 (4.37, 5.14) | 4.76 (4.36, 5.25) |
| IL6 | 4.76 (4.35, 5.29) | 4.89 (4.41, 5.50) | 4.95 (4.56, 5.54) | 5.32 (4.79, 5.96) | 4.91 (4.46, 5.52) |
| CSTB | 5.50 (5.18, 5.79) | 5.43 (5.12, 5.76) | 5.39 (5.08, 5.80) | 5.51 (5.27, 5.85) | 5.48 (5.17, 5.80) |
| MCP1 | 3.95 (3.53, 4.37) | 4.02 (3.59, 4.47) | 4.05 (3.66, 4.54) | 4.24 (3.74, 4.68) | 4.04 (3.60, 4.49) |
| KLK6 | 6.76 (6.52, 7.04) | 6.82 (±0.48) | 6.80 (6.50, 7.11) | 6.83 (±0.43) | 6.80 (6.52, 7.08) |
| Gal3 | 6.06 (±0.37) | 6.00 (±0.43) | 5.92 (5.64, 6.25) | 5.94 (5.65, 6.25) | 6.00 (5.73, 6.29) |
| PAR1 | 6.83 (6.49, 7.21) | 6.84 (6.52, 7.20) | 6.87 (6.53, 7.23) | 6.96 (6.70, 7.33) | 6.87 (6.54, 7.23) |
| TRAIL | 9.16 (8.80, 9.91) | 9.21 (8.83, 9.81) | 9.23 (8.84, 9.69) | 9.22 (8.93, 9.76) | 9.19 (8.84, 9.82) |
| hK11 | 5.21 (4.95, 5.46) | 5.29 (±0.52) | 5.35 (±0.51) | 5.48 (5.22, 5.75) | 5.29 (5.00, 5.61) |
| TIE2 | 6.54 (6.35, 6.73) | 6.57 (6.31, 6.82) | 6.57 (6.30, 6.84) | 6.59 (6.37, 6.78) | 6.56 (6.34, 6.78) |
| TF | 6.34 (6.06, 6.59) | 6.44 (6.12, 6.72) | 6.45 (6.11, 6.79) | 6.54 (±0.39) | 6.41 (6.12, 6.69) |
| TNFR1 | 12.62 (12.40, 12.85) | 12.66 (12.36, 12.98) | 12.69 (12.38, 13.05) | 12.82 (12.63, 13.07) | 12.68 (12.42, 12.95) |
| PDGFSubunitB | 6.81 (5.95, 7.85) | 6.75 (5.79, 7.54) | 6.80 (5.78, 7.56) | 6.62 (5.84, 7.38) | 6.77 (5.87, 7.62) |
| IL27A | 2.58 (±0.38) | 2.69 (±0.44) | 2.74 (±0.46) | 2.82 (±0.40) | 2.68 (±0.42) |
| CSF1 | 8.94 (±0.28) | 9.01 (8.77, 9.24) | 9.02 (8.77, 9.27) | 9.10 (±0.25) | 9.00 (8.80, 9.21) |
| CXCL1 | 6.09 (5.02, 7.24) | 6.29 (5.29, 7.25) | 6.66 (5.58, 7.35) | 6.68 (5.73, 7.50) | 6.36 (5.32, 7.31) |
| LOX1 | 4.68 (4.29, 5.07) | 4.53 (4.12, 5.10) | 4.58 (4.07, 5.01) | 4.56 (4.24, 4.92) | 4.61 (4.22, 5.03) |
| TRAILR2 | 1.70 (1.09, 2.00) | 1.77 (1.24, 2.18) | 1.90 (1.39, 2.29) | 2.04 (1.54, 2.31) | 1.80 (1.23, 2.14) |
| FGF23 | 2.84 (2.52, 3.17) | 2.92 (2.60, 3.24) | 2.97 (2.61, 3.32) | 3.00 (2.69, 3.34) | 2.91 (2.57, 3.25) |
| SCF | 7.42 (7.13, 7.67) | 7.44 (7.09, 7.71) | 7.39 (7.04, 7.72) | 7.42 (7.12, 7.68) | 7.42 (7.11, 7.69) |
| IL18 | 10.25 (9.90, 10.63) | 10.31 (9.90, 10.71) | 10.29 (9.89, 10.69) | 10.36 (10.05, 10.74) | 10.29 (9.92, 10.67) |
| IL6RA | 7.14 (6.82, 7.46) | 7.12 (6.79, 7.45) | 7.08 (±0.48) | 6.94 (6.65, 7.22) | 7.08 (6.76, 7.41) |
| TNFR2 | 5.28 (5.02, 5.53) | 5.32 (4.99, 5.70) | 5.37 (5.00, 5.76) | 5.46 (5.21, 5.79) | 5.34 (5.05, 5.64) |
| MMP3 | 0.84 (0.55, 1.24) | 1.07 (0.68, 1.46) | 1.18 (0.82, 1.60) | 1.41 (1.03, 1.75) | 1.06 (0.68, 1.48) |
| HSP27 | 2.21 (1.34, 3.74) | 1.97 (1.36, 3.13) | 1.97 (1.39, 3.03) | 2.09 (1.41, 3.29) | 2.09 (1.37, 3.33) |
| TNFSF14 | 2.32 (1.96, 2.73) | 2.23 (1.88, 2.64) | 2.16 (1.81, 2.61) | 2.24 (1.93, 2.57) | 2.26 (1.90, 2.66) |
| PRL | 4.18 (3.70, 4.67) | 4.15 (3.64, 4.63) | 4.10 (3.70, 4.61) | 4.19 (3.76, 4.71) | 4.16 (3.69, 4.66) |
| MPO | 3.69 (3.48, 3.89) | 3.69 (3.46, 3.90) | 3.67 (3.47, 3.92) | 3.72 (3.51, 3.93) | 3.70 (3.48, 3.90) |
| GH | 8.59 (6.96, 10.25) | 8.67 (6.86, 10.13) | 8.68 (7.05, 10.33) | 8.58 (6.86, 10.12) | 8.62 (6.95, 10.22) |
| MMP1 | 3.19 (2.51, 4.02) | 3.17 (2.47, 3.98) | 3.36 (±1.25) | 3.38 (±1.16) | 3.24 (2.52, 4.07) |
| RETN | 6.97 (6.62, 7.32) | 6.97 (6.59, 7.36) | 6.97 (6.58, 7.35) | 6.96 (6.67, 7.32) | 6.97 (6.62, 7.33) |
| FAS | 7.78 (7.56, 8.01) | 7.84 (7.54, 8.14) | 7.87 (7.58, 8.16) | 7.96 (7.76, 8.17) | 7.85 (7.60, 8.09) |
| PAPPA | 2.02 (1.68, 2.43) | 2.20 (±0.61) | 2.32 (±0.61) | 2.35 (1.94, 2.74) | 2.18 (1.78, 2.59) |
| PTX3 | 1.05 (0.79, 1.37) | 1.09 (±0.50) | 1.03 (0.66, 1.38) | 1.07 (±0.40) | 1.06 (0.76, 1.38) |
| REN | 7.13 (6.63, 7.72) | 7.41 (±0.94) | 7.61 (±0.97) | 7.93 (7.21, 8.63) | 7.36 (6.78, 8.07) |
| CHI3L1 | 6.18 (5.69, 6.76) | 6.28 (5.75, 6.94) | 6.39 (5.79, 7.06) | 6.51 (5.99, 7.14) | 6.30 (5.77, 6.93) |
| ST2 | 3.39 (3.06, 3.72) | 3.50 (±0.55) | 3.64 (3.29, 3.98) | 3.70 (3.39, 4.02) | 3.51 (3.17, 3.86) |
| TIM | 5.86 (5.39, 6.36) | 5.95 (5.50, 6.49) | 6.14 (5.63, 6.68) | 6.28 (5.77, 6.84) | 6.00 (5.51, 6.52) |
| BetaNGF | 0.82 (0.65, 1.02) | 0.89 (0.66, 1.08) | 0.88 (0.66, 1.12) | 0.90 (0.73, 1.08) | 0.86 (0.67, 1.06) |
| mAmP | 3.05 (2.30, 3.86) | 3.12 (2.24, 3.91) | 2.99 (1.99, 3.85) | 2.78 (1.82, 3.68) | 3.01 (2.14, 3.82) |
| TRANCE | 4.35 (3.92, 4.79) | 4.30 (±0.69) | 4.33 (±0.71) | 4.32 (±0.66) | 4.32 (±0.68) |
| HGF | 6.76 (6.53, 7.06) | 6.77 (6.46, 7.12) | 6.76 (6.49, 7.19) | 6.88 (6.66, 7.18) | 6.79 (6.53, 7.12) |
| PSGL1 | 0.87 (0.67, 1.08) | 0.95 (0.70, 1.13) | 0.90 (0.66, 1.16) | 0.99 (±0.29) | 0.92 (0.69, 1.13) |
| MB | 5.38 (5.04, 5.77) | 5.47 (5.11, 5.93) | 5.59 (5.23, 6.03) | 5.71 (5.36, 6.11) | 5.49 (5.13, 5.91) |
| TM | 9.93 (±0.38) | 10.00 (9.70, 10.31) | 10.03 (±0.45) | 10.12 (±0.34) | 10.00 (9.71, 10.28) |
| IL16 | 4.21 (±0.47) | 4.30 (3.89, 4.65) | 4.31 (±0.56) | 4.43 (±0.42) | 4.29 (3.94, 4.63) |
| MMP10 | 6.52 (6.15, 6.94) | 6.58 (6.15, 7.06) | 6.55 (6.15, 7.03) | 6.74 (6.36, 7.12) | 6.58 (6.19, 7.02) |
| UPAR | 10.16 (±0.30) | 10.18 (±0.40) | 10.22 (9.87, 10.50) | 10.29 (±0.29) | 10.19 (±0.35) |
| CCL4 | 7.53 (7.21, 7.96) | 7.52 (7.18, 7.96) | 7.63 (7.20, 8.05) | 7.67 (7.35, 8.06) | 7.57 (7.22, 7.99) |
| CTSD | 7.43 (7.14, 7.76) | 7.44 (7.09, 7.80) | 7.49 (±0.53) | 7.55 (7.26, 7.91) | 7.46 (7.15, 7.81) |
| RAGE | 4.70 (4.41, 4.94) | 4.61 (±0.48) | 4.56 (±0.51) | 4.55 (±0.42) | 4.64 (4.31, 4.93) |
| CCL3 | 2.37 (2.00, 2.75) | 2.36 (2.00, 2.73) | 2.35 (1.98, 2.72) | 2.41 (±0.43) | 2.36 (2.02, 2.74) |
| MMP7 | 6.99 (5.54, 8.03) | 7.42 (5.93, 8.42) | 7.60 (5.98, 8.54) | 7.74 (5.87, 8.74) | 7.34 (5.76, 8.37) |
| CXCL6 | 5.98 (5.35, 6.67) | 6.05 (5.50, 6.59) | 6.18 (±0.89) | 6.27 (±0.84) | 6.08 (5.49, 6.70) |
| ITGB1BP2 | 1.74 (1.05, 3.04) | 1.63 (1.06, 2.52) | 1.62 (1.08, 2.31) | 1.69 (1.14, 2.70) | 1.68 (1.07, 2.72) |
| CXCL16 | 3.81 (3.59, 4.01) | 3.82 (3.57, 4.07) | 3.84 (3.59, 4.07) | 3.84 (3.64, 4.06) | 3.82 (3.59, 4.05) |
| Dkk1 | 5.32 (4.82, 5.90) | 5.27 (4.74, 5.76) | 5.33 (4.82, 5.81) | 5.24 (±0.68) | 5.29 (4.79, 5.81) |
| SIRT2 | 2.52 (1.72, 3.75) | 2.20 (1.54, 3.23) | 2.02 (1.48, 3.02) | 2.13 (1.55, 3.19) | 2.29 (1.59, 3.37) |
| GAL | 5.33 (4.91, 5.82) | 5.31 (4.84, 5.78) | 5.34 (±0.78) | 5.36 (±0.69) | 5.33 (4.87, 5.82) |
| AGRP | 4.36 (4.11, 4.64) | 4.36 (4.01, 4.74) | 4.33 (3.94, 4.74) | 4.34 (4.04, 4.65) | 4.35 (4.05, 4.68) |
| ENRAGE | 1.07 (0.71, 1.46) | 1.09 (0.69, 1.55) | 1.12 (0.72, 1.53) | 1.20 (0.85, 1.67) | 1.11 (0.74, 1.53) |
| CD40 | 9.17 (8.84, 9.65) | 9.20 (8.89, 9.56) | 9.22 (8.88, 9.57) | 9.26 (8.95, 9.64) | 9.20 (8.88, 9.61) |
| tPA | 7.78 (7.39, 8.27) | 7.80 (7.39, 8.22) | 7.80 (7.42, 8.13) | 7.72 (7.45, 8.13) | 7.78 (7.40, 8.22) |
| HBEGF | 4.66 (4.44, 5.01) | 4.65 (4.42, 4.90) | 4.64 (4.43, 4.95) | 4.66 (4.46, 4.90) | 4.65 (4.44, 4.94) |
| ESM1 | 3.74 (±0.43) | 3.79 (±0.46) | 3.83 (3.50, 4.17) | 3.84 (3.58, 4.18) | 3.77 (3.49, 4.09) |
| IL4 | -0.09 (-0.37, 0.19) | -0.05 (±0.48) | -0.03 (-0.33, 0.24) | -0.02 (-0.35, 0.22) | -0.06 (-0.36, 0.21) |
| VEGFD | 6.87 (6.55, 7.17) | 6.94 (6.58, 7.31) | 6.93 (6.59, 7.30) | 6.99 (6.71, 7.31) | 6.92 (6.59, 7.25) |
| MMP12 | 6.87 (±0.60) | 7.09 (±0.64) | 7.32 (6.87, 7.79) | 7.49 (7.06, 7.97) | 7.10 (6.64, 7.56) |
| SPON1 | 4.88 (4.66, 5.09) | 4.94 (4.66, 5.20) | 4.96 (4.68, 5.25) | 5.01 (4.82, 5.22) | 4.93 (4.70, 5.16) |
| CASP8 | 1.15 (0.77, 1.70) | 1.06 (0.75, 1.47) | 1.02 (0.69, 1.43) | 1.15 (0.82, 1.48) | 1.10 (0.76, 1.57) |
| CTSL1 | 5.87 (5.60, 6.16) | 5.95 (±0.48) | 5.98 (5.71, 6.35) | 6.13 (5.90, 6.42) | 5.98 (5.67, 6.27) |
| CX3CL1 | 5.23 (±0.51) | 5.41 (5.02, 5.78) | 5.50 (±0.60) | 5.61 (±0.48) | 5.38 (±0.55) |
| FABP4 | 3.23 (2.81, 3.60) | 3.09 (±0.64) | 2.93 (2.44, 3.41) | 2.99 (±0.61) | 3.09 (2.66, 3.53) |
| BNP | 0.91 (0.49, 1.41) | 1.02 (0.62, 1.65) | 1.15 (0.63, 1.90) | 1.30 (0.74, 2.25) | 1.03 (0.59, 1.68) |
| LEP | 3.48 (2.76, 4.04) | 3.14 (2.44, 3.80) | 2.87 (±1.06) | 2.83 (±0.98) | 3.17 (2.46, 3.85) |
| CCL20 | 6.02 (5.45, 6.64) | 6.00 (5.45, 6.63) | 6.09 (5.53, 6.73) | 6.00 (5.53, 6.75) | 6.02 (5.47, 6.67) |
| CA125 | 5.05 (4.48, 5.57) | 5.19 (4.61, 5.72) | 5.27 (±0.84) | 5.47 (±0.86) | 5.19 (4.61, 5.72) |
| NEMO | 2.72 (1.89, 4.02) | 2.55 (1.94, 3.63) | 2.60 (2.01, 3.50) | 2.75 (2.13, 3.81) | 2.66 (1.96, 3.79) |
| FS | 5.47 (±0.50) | 5.50 (±0.52) | 5.54 (±0.53) | 5.60 (±0.49) | 5.50 (5.17, 5.85) |
| PECAM1 | 5.91 (5.64, 6.23) | 5.95 (5.67, 6.22) | 5.94 (5.66, 6.24) | 6.04 (5.77, 6.30) | 5.95 (5.67, 6.24) |
| ECP | 5.94 (5.51, 6.34) | 5.92 (5.44, 6.31) | 5.85 (5.41, 6.25) | 5.87 (5.55, 6.26) | 5.91 (5.49, 6.31) |
| NTproBNP | 3.50 (±1.00) | 3.73 (±1.04) | 3.99 (3.07, 4.72) | 4.13 (3.40, 4.94) | 3.75 (3.00, 4.45) |

Continuous variables were reported as mean (±SD) if Shapiro-Wilk test with P-value ≥0.05 and as median (25% percentage, 75% percentage) if Shapiro-Wilk test with P-value <0.05. Categorical variables are reported as number and percentage.

### **Table S3.** Contingency table showing the 3-year ASCVD risk reclassification improvement by NRI adding the original endotypes to SCORE2 in the IMPROVE derived dataset.

| **Pr (SCORE2)** | **Pr (SCORE2 + endotype)** | | | **NRI (95%CI)** |
| --- | --- | --- | --- | --- |
|  | **< 0.05** | **< 0.1** | **>= 0.1** |  |
| **Overall** |  |  |  |  |
| <0.05 | 1683 | 284 | 0 | **0.150 (0.026-0.296)** |
| <0.1 | 345 | 544 | 98 |  |
| >=0.1 | 0 | 15 | 152 |  |
| **Event** |  |  |  |  |
| <0.05 | 45 | 22 | 0 | **0.131 (0.001-0.310)** |
| <0.1 | 15 | 36 | 14 |  |
| >=0.1 | 0 | 1 | 20 |  |
| **Non-event** |  |  |  |  |
| <0.05 | 1207 | 160 | 0 | 0.019 (-0.068-0.113) |
| <0.1 | 238 | 305 | 48 |  |
| >=0.1 | 0 | 9 | 75 |  |

Overall refers to all the participants in the derived dataset. Event refers to participants with ASCVD (n=166) within 3 years. Non-event refers to participants without ASCVD within 3 years (n= 2955).

SCORE2 was calculated by the public available coefficient generated by SCORE2 working group and ESC Cardiovascular risk collaboration, and detail of coefficient were shown in SCORE2 Updated Supplementary Material, Supplementary methods Table 2 in the original SCORE2 paper. *^34^*

For calculating Pr (SCORE2), i.e the probability of 3-year ASCVD using SCORE2, we incorporated SCORE2 in Cox regression model to reweight the coefficient and adapt the model to predict a 3-year ASCVD risk and achieve a better discriminative capacity. To calculate Pr (SCORE2 + endotype), in the above regression model, we added endotype as an independent variable.

NRI: Net Reclassification Improvement

CI: Confidence Interval

ASCVD: Atherosclerotic Cardiovascular Disease

### **Table S4.** Contingency table showing the 3-year ASCVD risk reclassification improvement by NRI adding the original endotype to c-IMT_mean-max_ in IMPROVE derived dataset.

| **c-IMT_mean-max_** | **c-IMT_mean-max_ + Endotype** | | | **NRI (95%CI)** |
| --- | --- | --- | --- | --- |
|  | **< 0.05** | **< 0.1** | **>= 0.1** |  |
| **Overall** |  |  |  |  |
| <0.05 | 1600 | 389 | 0 | **0.121 (0.008-0.330)** |
| <0.1 | 425 | 418 | 142 |  |
| >=0.1 | 0 | 22 | 125 |  |
| **Event** |  |  |  |  |
| <0.05 | 40 | 22 | 0 | 0.113 (-0.009-0.340) |
| <0.1 | 17 | 46 | 14 |  |
| >=0.1 | 0 | 2 | 12 |  |
| **Non-event** |  |  |  |  |
| <0.05 | 1152 | 218 | 0 | 0.008 (-0.077-0.108) |
| <0.1 | 294 | 226 | 72 |  |
| >=0.1 | 0 | 13 | 67 |  |

Overall refers to all the participants in IMPROVE. Event refers to participants with ASCVD within 3 years. Non-event refers to participants without ASCVD within 3 years.

For calculating probability of event using c-IMT_mean-max_, Pr(c-IMT_mean-max_), we incorporated c-IMT_mean-max_ in cox regression to model the ASCVD risk within 3 years in IMPROVE. For calculating Pr (c-IMT_mean-max_ + Endotype), we added endotype as an independent variable.

NRI: Net Reclassification Improvement

CI: Confidence Interval

ASCVD: Atherosclerotic Cardiovascular Disease

### **Table S5.** Contingency table showing the 3-year ASCVD risk reclassification improvement by NRI adding the predicted endotypes to the presence of carotid plaque in IMPROVE derived dataset.

| **Pr(Plaque)** | **Pr(Plaque + Endotype)** | | | **NRI (95%CI)** |
| --- | --- | --- | --- | --- |
|  | **< 0.05** | **< 0.1** | **>= 0.1** |  |
| **Overall** |  |  |  |  |
| <0.05 | 929 | 47 | 0 | **0.321 (0.048-0.548)** |
| <0.1 | 1210 | 422 | 510 |  |
| >=0.1 | 0 | 0 | 0 |  |
| **Event** |  |  |  |  |
| <0.05 | 21 | 4 | 0 | 0.045 (-0.341-0.418) |
| <0.1 | 44 | 37 | 47 |  |
| >=0.1 | 0 | 0 | 0 |  |
| **Non-event** |  |  |  |  |
| <0.05 | 660 | 23 | 0 | **0.276 (0.037-0.429)** |
| <0.1 | 853 | 238 | 267 |  |
| >=0.1 | 0 | 0 | 0 |  |

Overall refers to all the participants in IMPROVE. Event refers to participants with ASCVD within 3 years. Non-event refers to participants without ASCVD within 3 years.

For calculating probability of event using plaque, Pr(plaque), we incorporated plaque in cox regression to model the ASCVD within 3 years in IMPROVE. For calculating Pr (plaque + Endotype), in the above regression model specification, we added endotype as an independent variable.

NRI: Net Reclassification Improvement

CI: Confidence Interval

ASCVD: Atherosclerotic Cardiovascular Disease

### **Table S6.** Distribution of the carotid ultrasonographic measures and proportion of ASCVD across the 4 predicted endotypes in the IMPROVE derived dataset (n=3121).

|  | **Predicted endotype 1** | **Predicted endotype 2** | **Predicted endotype 3** | **Predicted endotype 4** | **Overall** |
| --- | --- | --- | --- | --- | --- |
|  | **(N=1521)** | **(N=521)** | **(N=529)** | **(N=550)** | **(N=3121)** |
| ***Ultrasonographic measures (mm)*** | | |  |  |  |
| **c-IMT_mean_** | 0.81 (±0.15) | 0.89 (±0.18) | 0.96 (±0.20) | 1.04 (±0.22) | 0.89 (±0.20) |
| **c-IMT_max_** | 1.74 (±0.66) | 2.06 (±0.74) | 2.26 (±0.81) | 2.57 (±0.89) | 2.03 (±0.81) |
| **c-IMT_mean-max_** | 1.12 (±0.22) | 1.26 (±0.27) | 1.36 (±0.29) | 1.48 (±0.33) | 1.25 (±0.30) |
| **Number of plaques** | 0.99 (±1.19) | 1.63 (±1.47) | 2.08 (±1.63) | 2.60 (±1.65) | 1.56 (±1.54) |
| **Area of plaques_bulb_** | 22.60 (±28.90) | 39.26 (±38.97) | 51.87 (±46.34) | 67.07 (±48.85) | 38.15 (±41.71) |
| **Area of plaques_no-bulb_** | 8.55 (±17.36) | 17.18 (±26.34) | 24.98 (±35.16) | 34.44 (±36.75) | 17.33 (±28.43) |
| **ICCAD_mean_** | 7.50 (±0.71) | 7.81 (±0.74) | 8.13 (±0.83) | 8.46 (±0.88) | 7.83 (±0.85) |
| **c-IMT_mean-max-progr_** | 0.02 (±0.05) | 0.02 (±0.05) | 0.03 (±0.06) | 0.03 (±0.06) | 0.02 (±0.05) |
| **c-IMT_-fastest-progr_** | 0.14 (±0.13) | 0.16 (±0.13) | 0.19 (±0.16) | 0.22 (±0.17) | 0.17 (±0.14) |
| **Area of plaques_bulb-progr_** | 2.17 (±7.65) | 3.13 (±7.98) | 3.43 (±9.80) | 4.87 (±10.94) | 2.99 (±8.76) |
| **Area of plaques_no-bulb-progr_** | 0.94 (±5.23) | 1.59 (±6.14) | 2.13 (±7.80) | 2.97 (±8.79) | 1.59 (±6.60) |
| **ICCAD_mean_** change over time | 0.00 (±0.03) | 0.00 (±0.03) | 0.01 (±0.03) | 0.01 (±0.03) | 0.00 (±0.03) |
| ***Survival outcome n(%)*** | |  |  |  |  |
| **ASCVD** | |  |  |  |  |
| Yes | 45 (3.0) | 23 (4.4) | 43 (8.1) | 55 (10.0) | 166 (5.3) |
| No | 1476 (97.0) | 498 (95.6) | 486 (91.9) | 495 (90.0) | 2955 (94.7) |
| **Cardiac events** | |  |  |  |  |
| Yes | 27 (1.8) | 16 (3.1) | 24 (4.5) | 36 (6.5) | 103 (3.3) |
| No | 1494 (98.2) | 505 (96.9) | 505 (95.5) | 514 (93.5) | 3018 (96.7) |
| **Cerebrovascular events** | | |  |  |  |
| Yes | 16 (1.1) | 7 (1.3) | 16 (3.0) | 17 (3.1) | 56 (1.8) |
| No | 1505 (98.9) | 514 (98.7) | 513 (97.0) | 533 (96.9) | 3065 (98.2) |
| **Peripheral events** | |  |  |  |  |
| Yes | 2 (0.1) | 0 (0) | 3 (0.6) | 2 (0.4) | 7 (0.2) |
| No | 1519 (99.9) | 521 (100) | 526 (99.4) | 548 (99.6) | 3114 (99.8) |

Continuous variables were reported as mean (±standard deviation), and categorical variables was as frequency (percentage, %). Missing values are reported in the footnote of Table 1.

Predicted endotype refers to the endotypes predicted by the stacking model

ASCVD: Atherosclerotic Cardiovascular Disease

### **Table S7.** Distribution of the ultrasonographic measures across the 4 predicted endotypes in the IMPROVE replicated dataset (n=219).

|  | **Predicted endotype 1** | **Predicted endotype 2** | **Predicted endotype 3** | **Predicted endotype 4** | **Overall** |
| --- | --- | --- | --- | --- | --- |
|  | **(N=112)** | **(N=52)** | **(N=21)** | **(N=34)** | **(N=219)** |
| ***Ultrasonographic measures (mm)*** | |  |  |  |  |
| **c-IMT_Mean_** | 0.80 (±0.16) | 0.89 (±0.18) | 0.98 (±0.22) | 1.04 (±0.24) | 0.88 (±0.20) |
| **c-IMT_max_** | 1.79 (±0.75) | 2.06 (±0.72) | 2.28 (±0.86) | 2.50 (±0.95) | 2.01 (±0.83) |
| **c-IMT_mean-max_** | 1.12 (±0.24) | 1.24 (±0.23) | 1.37 (±0.31) | 1.46 (±0.35) | 1.23 (±0.29) |
| **Area of plaques_bulb_** | 23.44 (±31.16) | 39.76 (±35.72) | 57.08 (±62.69) | 66.40 (±54.45) | 37.21 (±43.27) |
| **Area of plaques_no-bulb_** | 9.72 (±19.64) | 16.92 (±25.59) | 32.16 (±49.14) | 37.26 (±43.27) | 17.86 (±31.10) |
| **Number of plaques** | 1.00 (±1.26) | 1.73 (±1.47) | 2.24 (±2.02) | 2.50 (±1.78) | 1.53 (±1.59) |
| **ICCAD_mean_** | 7.38 (±0.68) | 7.81 (±0.84) | 8.29 (±0.94) | 8.48 (±0.86) | 7.74 (±0.88) |
| **c-IMT_mean-max-progr_** | 0.02 (±0.04) | 0.03 (±0.07) | 0.04 (±0.06) | 0.05 (±0.07) | 0.03 (±0.06) |
| Missing n(%) | 19 (17.0) | 10 (19.2) | 4 (19.0) | 10 (29.4) | 43 (19.6) |
| **c-IMT _fastest-progr_** | 0.14 (±0.12) | 0.19 (±0.16) | 0.21 (±0.18) | 0.22 (±0.16) | 0.17 (±0.15) |
| Missing n(%) | 19 (17.0) | 10 (19.2) | 4 (19.0) | 10 (29.4) | 43 (19.6) |
| **Area of plaques_bulb-progr_** | 2.24 (±5.59) | 2.38 (±9.39) | 4.98 (±9.83) | 4.78 (±9.15) | 2.88 (±7.6) |
| Missing n(%) | 20 (17.9) | 10 (19.2) | 4 (19.0) | 11 (32.4) | 45 (20.5) |
| **Area of plaques_no-bulb-progr_** | 1.09 (±3.62) | 1.35 (±7.64) | 1.99 (±7.46) | 2.07 (±8.19) | 1.37 (±5.88) |
| Missing n(%) | 20 (17.9) | 10 (19.2) | 4 (19.0) | 11 (32.4) | 45 (20.5) |
| **ICCAD_-mean_** change over time | 0.00 (±0.03) | 0.01 (±0.03) | 0.01 (±0.02) | -0.00 (±0.05) | 0.00 (±0.03) |
| Missing n(%) | 19 (17.0) | 10 (19.2) | 4 (19.0) | 10 (29.4) | 43 (19.6) |

Continuous variable was reported as mean (±standard deviation), and categorical variable was reported as frequency (percentage %). Missing values are reported for each variable as n (%).

### **Table S8.** Association between predicted endotypes and atherosclerosis-related outcome in the IMPROVE derived dataset (n=3121).

|  | **Model 1** | | **Model 2** | | **Model 3** | |
| --- | --- | --- | --- | --- | --- | --- |
|  | **β (SE)** | **P-value** | **β (SE)** | **P-value** | **β (SE)** | **P-value** |
| **c-IMT_mean_** |  |  |  |  |  |  |
| Predicted Endotype 2 | 0.079 (0.009) | <0.0001 | - | **-** | - | **-** |
| Predicted Endotype 3 | 0.141 (0.009) | <0.0001 | - | **-** | - | **-** |
| Predicted Endotype 4 | 0.213 (0.009) | <0.0001 | - | **-** | - | **-** |
| **c-IMT_mean-max_** |  |  |  |  |  |  |
| Predicted Endotype 2 | 0.121 (0.013) | <0.0001 | - | **-** | - | **-** |
| Predicted Endotype 3 | 0.203 (0.014) | <0.0001 | - | **-** | - | **-** |
| Predicted Endotype 4 | 0.324 (0.014) | <0.0001 | - | **-** | - | **-** |
| **c-IMT_max_** |  |  |  |  |  |  |
| Predicted Endotype 2 | 0.293 (0.038) | <0.0001 | - | **-** | - | **-** |
| Predicted Endotype 3 | 0.46 (0.039) | <0.0001 | - | **-** | - | **-** |
| Predicted Endotype 4 | 0.765 (0.039) | <0.0001 | - | **-** | - | **-** |
| **Number of plaques** |  |  |  |  |  |  |
| Predicted Endotype 2 | 0.585 (0.072) | <0.0001 | - | **-** | - | **-** |
| Predicted Endotype 3 | 0.972 (0.074) | <0.0001 | - | **-** | - | **-** |
| Predicted Endotype 4 | 1.473 (0.074) | <0.0001 | - | **-** | - | **-** |
| **Area of plaques_bulb_** |  |  |  |  |  |  |
| Predicted Endotype 2 | 15.21 (1.94) | <0.0001 | - | **-** | - | **-** |
| Predicted Endotype 3 | 26.38 (2.00) | <0.0001 | - | **-** | - | **-** |
| Predicted Endotype 4 | 41.17 (2.01) | <0.0001 | - | **-** | - | **-** |
| **Area of plaques_no-bulb_** |  |  |  |  |  |  |
| Predicted Endotype 2 | 8.08 (1.37) | <0.0001 | - | **-** | - | **-** |
| Predicted Endotype 3 | 15.4 (1.41) | <0.0001 | - | **-** | - | **-** |
| Predicted Endotype 4 | 24.7 (1.41) | <0.0001 | - | **-** | - | **-** |
| I**CCAD_mean_** |  |  |  |  |  |  |
| Predicted Endotype 2 | 0.26 (0.039) | <0.0001 | - | **-** | - | **-** |
| Predicted Endotype 3 | 0.54 (0.040) | <0.0001 | - | **-** | - | **-** |
| Predicted Endotype 4 | 0.86 (0.040) | <0.0001 | - | **-** | - | **-** |
| **c-IMT_mean-max-progr_** |  |  |  |  |  |  |
| Predicted Endotype 2 | -0.001 (0.003) | 0.82 | 0.002 (0.003) | 0.41 | 0.002 (0.003) | 0.42 |
| Predicted Endotype 3 | 0.005 (0.003) | 0.07 | 0.010 (0.003) | 0.001 | 0.010 (0.003) | 0.001 |
| Predicted Endotype 4 | 0.01 (0.003) | 0.001 | 0.018 (0.003) | <0.0001 | 0.018 (0.003) | <0.0001 |
| **Log_10_(c-IMT_fastest-prog_+ 0.1)** |  |  |  |  |  |  |
| Predicted Endotype 2 | 0.03 (0.011) | 0.003 | 0.018 (0.011) | 0.09 | 0.017 (0.011) | 0.11 |
| Predicted Endotype 3 | 0.05 (0.011) | <0.0001 | 0.028 (0.011) | 0.01 | 0.029 (0.011) | 0.01 |
| Predicted Endotype 4 | 0.10 (0.011) | <0.0001 | 0.061 (0.012) | <0.0001 | 0.060 (0.012) | <0.0001 |
| **Area of plaques_bulb-progr_** |  |  |  |  |  |  |
| Predicted Endotype 2 | 0.90 (0.47) | 0.05 | 1.39 (0.47) | 0.003 | 1.37 (0.47) | 0.004 |
| Predicted Endotype 3 | 1.14 (0.49) | 0.02 | 2.06 (0.50) | <0.0001 | 2.04 (0.50) | <0.0001 |
| Predicted Endotype 4 | 2.56 (0.49) | <0.0001 | 3.98 (0.53) | <0.0001 | 3.97 (0.53) | <0.0001 |
| **Area of plaques_no-bulb-progr_** |  |  |  |  |  |  |
| Predicted Endotype 2 | 0.65 (0.35) | 0.07 | 0.93 (0.35) | 0.008 | 0.92 (0.35) | 0.009 |
| Predicted Endotype 3 | 1.17 (0.37) | 0.001 | 1.77 (0.37) | <0.0001 | 1.75 (0.37) | <0.0001 |
| Predicted Endotype 4 | 2.03 (0.37) | <0.0001 | 2.92 (0.39) | <0.0001 | 2.92 (0.39) | <0.0001 |
| **ICCAD_mean_ change over time** |  |  |  |  |  |  |
| Predicted Endotype 2 | 0.001 (0.002) | 0.47 | 0.002 (0.002) | 0.21 | 0.002 (0.002) | 0.22 |
| Predicted Endotype 3 | 0.002 (0.002) | 0.33 | 0.003 (0.002) | 0.08 | 0.003 (0.002) | 0.09 |
| Predicted Endotype 4 | 0.001 (0.002) | 0.57 | 0.003 (0.002) | 0.08 | 0.003 (0.002) | 0.10 |
|  | **HR (95%CI)** | **P-value** | **HR (95%CI)** | **P-value** | **HR (95%CI)** | **P-value** |
| **ASCVD** |  |  |  |  |  |  |
| Predicted Endotype 2 | 1.42 (0.85-2.36) | 0.17 | 1.28 (0.77-2.14) | 0.34 | 1.30 (0.78-2.17) | 0.31 |
| Predicted Endotype 3 | 2.55 (1.65-3.95) | <0.0001 | 2.19 (1.37-3.46) | 0.001 | 2.24 (1.42-3.53) | 0.001 |
| Predicted Endotype 4 | 3.45 (2.26-5.26) | <0.0001 | 2.60 (1.63-4.15) | <0.0001 | 2.54 (1.58-4.07) | <0.0001 |
| **Cardiac events** |  |  |  |  |  |  |
| Predicted Endotype 2 | 1.69 (0.92-3.12) | 0.09 | 1.55 (0.83-2.87) | 0.16 | 1.62 (0.87-3.01) | 0.13 |
| Predicted Endotype 3 | 2.47 (1.41-4.33) | 0.002 | 2.26 (1.26-4.05) | 0.006 | 2.38 (1.32-4.29) | 0.004 |
| Predicted Endotype 4 | 3.64 (2.14-6.18) | <0.0001 | 2.94 (1.64-5.27) | <0.0001 | 2.90 (1.61-5.25) | <0.0001 |
| **Cerebrovascular events** |  |  |  |  |  |  |
| Predicted Endotype 2 | 1.26 (0.51-3.09) | 0.61 | 1.09 (0.44-2.70) | 0.85 | 1.11 (0.45-2.74) | 0.82 |
| Predicted Endotype 3 | 2.93 (1.43-6.00) | 0.003 | 2.26 (1.07-4.77) | 0.03 | 2.33 (1.10-4.92) | 0.03 |
| Predicted Endotype 4 | 3.52 (1.72-7.22) | 0.001 | 2.34 (1.06-5.17) | 0.03 | 2.31 (1.04-5.15) | 0.04 |

Association between endotypes and ultrasonographic measures is presented as β coefficient (standard error). The association between endotypes and ASCVD is presented as HR (95% CI).

Model 1: adjusted Olink analytical batch, latitude; Model 2: model1+ diabetes mellitus, LDL-cholesterol, smoking, body mass index, and ultrasonographic measures at baseline; For the regression for Log10 (c-IMT_fastest-progr_ + 0.1) adjusted ultrasound measure of c-IMT_max_, and also the regression for ICCAD_mean_ change over time adjusted ICCAD_mean_. The other model adjusted c-IMT_mean-max_; Model 3: model 2 + anti-platelet treatment + anti-hypertensive treatment + lipid-lowering treatment

### **Table S9.** Contingency table showing the 3-year ASCVD risk reclassification improvement by NRI using the predicted endotype with reference to the SCORE2 in IMPROVE derived dataset.

| **Pr (SCORE2)** | **Pr (SCORE2 + Pred endotype)** | | | **NRI (95%CI)** |
| --- | --- | --- | --- | --- |
|  | **< 0.05** | **< 0.1** | **>= 0.1** |  |
| **Overall** |  |  |  |  |
| <0.05 | 1671 | 296 | 0 | **0.173 (0.044-0.306)** |
| <0.1 | 304 | 569 | 114 |  |
| >=0.1 | 4 | 11 | 152 |  |
| **Event** |  |  |  |  |
| <0.05 | 41 | 26 | 0 | **0.170 (0.036-0.364)** |
| <0.1 | 13 | 39 | 13 |  |
| >=0.1 | 0 | 0 | 21 |  |
| **Non-event** |  |  |  |  |
| <0.05 | 1207 | 160 | 0 | 0.003 (-0.120-0.105) |
| <0.1 | 212 | 323 | 56 |  |
| >=0.1 | 4 | 7 | 73 |  |

Overall refers to all the participants in derived cohort of IMPROVE. Event refers to participants with ASCVD within 3 years. Non-event refers to participants without ASCVD within 3 years.

SCORE2 was calculated by the public available coefficient generated by SCORE2 working group and ESC Cardiovascular risk collaboration, and detail of coefficient were shown in SCORE2 Updated Supplementary Material, Supplementary methods Table 2 for original SCORE2 paper. *^34^*

For calculating probability of event using SCORE2, Pr (SCORE2), we incorporated SCORE2 in cox regression model which reweighted the coefficient for adapting 3-years ASCVD to achieve better discrimination capacity. For calculating Pr (SCORE2 + predicted endotype), in the above regression model specification, we added endotype as an independent variable.

Pred endotype: predicted endotype

NRI: Net Reclassification Improvement

CI: Confidence Interval

ASCVD: Atherosclerotic Cardiovascular Disease

### **Table S10.** Distribution of the ultrasonographic measures and occurrence myocardial infarction and ischemic stroke across the 4 predicted endotypes in the PIVUS study.

|  | **Predicted endotype 1** | **Predicted endotype 2** | **Predicted endotype 3** | **Predicted endotype 4** | **Overall** |
| --- | --- | --- | --- | --- | --- |
|  | **(N=254)** | **(N=272)** | **(N=154)** | **(N=280)** | **(N=960)** |
| ***Ultrasonographic measures*** | | |  |  |  |
| c-IMT_mean_ (mm) | 0.86 (±0.15) | 0.86 (±0.15) | 0.90 (±0.19) | 0.93 (±0.17) | 0.89 (±0.17) |
| Area of plaque (mm2) | 9.37 (±13.51) | 11.73 (±16.02) | 15.46 (±17.23) | 21.11 (±18.82) | 14.45 (±17.12) |
| Presence of plaque (yes, n, %) | 119 (46.9) | 147 (54.0) | 97 (63.0) | 216 (77.1) | 579 (60.3) |
| ***Composite event (MI or IS)*** | 32 (12.6) | 50 (18.4) | 38 (24.7) | 96 (34.3) | 216 (22.5) |
| Myocardial infarction | 22 (8.7) | 24 (8.8) | 22 (14.3) | 72 (25.7) | 140 (14.6) |
| Ischemic stroke | 16 (6.3) | 26 (9.6) | 21 (13.6) | 38 (13.6) | 101 (10.5) |

### **Table S11.** Contingency table showing the 5-years ASCVD risk reclassification improvement adding the predicted endotypes to SCORE2-OP in PIVUS

| **Pr(SCORE2-OP)** | **Pr(SCORE2-OP + Pred endotype)** | | | **NRI (95%CI)** |
| --- | --- | --- | --- | --- |
|  | **< 0.075** | **< 0.15** | **>= 0.15** |  |
| Overall | |  |  |  |
| <0.075 | 598 | 103 | 0 | 0.096 (-0.013-0.324) |
| <0.15 | 57 | 104 | 15 |  |
| >=0.15 | 0 | 5 | 7 |  |
| Event |  |  |  |  |
| <0.075 | 27 | 7 | 0 | **0.153 (0.023-0.411)** |
| <0.15 | 1 | 10 | 3 |  |
| >=0.15 | 0 | 1 | 3 |  |
| Non-event | |  |  |  |
| <0.075 | 547 | 93 | 0 | -0.057 (-0.128-0.001) |
| <0.15 | 55 | 89 | 12 |  |
| >=0.15 | 0 | 4 | 4 |  |

Overall refers to all the participants in PIVUS. Event refers to participants with myocardial infarction or ischemic stroke within 5 years. Non-event refers to participants without myocardial infarction or ischemic stroke within 5 years.

SCORE2-OP was calculated by the public available coefficient generated by SCORE2-OP working group and ESC Cardiovascular risk collaboration, and detail of coefficient were shown in SCORE2-OP original publication. *^33^*

For calculating probability of event using SCORE2-OP, Pr (SCORE2-OP), we incorporated SCORE2-OP in cox regression model which reweighted the coefficient for adapting 5-years ASCVD for PIVUS cohort to achieve better discrimination capacity. For calculating Pr (SCORE2 + pred endotype), in the above regression model specification, we added endotype as an independent variable.

NRI: Net Reclassification Improvement

CI: Confidence Interval

ASCVD: Atherosclerotic Cardiovascular Disease

### **Table S12.** Contingency table showing the 10-year ASCVD risk reclassification improvement by NRI adding the predicted endotypes to SCORE2-OP in PIVUS.

| **Pr(SCORE2-OP)** | **Pr(SCORE2-OP + Pred endotype)** | | | **NRI (95%CI)** |
| --- | --- | --- | --- | --- |
|  | **< 0.075** | **< 0.15** | **>= 0.15** |  |
| All participants | |  |  |  |
| <0.075 | 112 | 25 | 0 | **0.233 (0.084-0.443)** |
| <0.15 | 144 | 294 | 104 |  |
| >=0.15 | 0 | 66 | 144 |  |
| Event |  |  |  |  |
| <0.075 | 8 | 2 | 0 | **0.095 (0.002-0.292)** |
| <0.15 | 6 | 30 | 20 |  |
| >=0.15 | 0 | 6 | 32 |  |
| Non-event | |  |  |  |
| <0.075 | 88 | 16 | 0 | **0.137 (0.034-0.217)** |
| <0.15 | 126 | 234 | 70 |  |
| >=0.15 | 0 | 54 | 96 |  |

Overall refers to all the participants in PIVUS. Event refers to participants with myocardial infarction or ischemic stroke within 10 years. Non-event refers to participants without myocardial infarction or ischemic stroke within 10 years.

SCORE2-OP was calculated by the public available coefficient generated by SCORE2-OP working group and ESC Cardiovascular risk collaboration, and detail of coefficient were shown in SCORE2-OP original publication.*^33^*

For calculating probability of event using SCORE2-OP, Pr (SCORE2-OP), we incorporated SCORE2-OP in cox regression model which reweighted the coefficient for adapting 10-years ASCVD for PIVUS cohort to achieve better discrimination capacity. For calculating Pr (SCORE2 + pred endotype), in the above regression model specification, we added endotype as an independent variable.

Pred endotype: predicted endotype

NRI: Net Reclassification Improvement

CI: Confidence Interval

ASCVD: Atherosclerotic Cardiovascular Disease (only myocardial infarction and ischemic stroke recorded in PIVUS)

### **Table S13.** Distribution of high-risk plaques and absence of CHD estimated by coronary CT angiography (CCTA) across the 4 predicted endotypes in the PACIFIC dataset.

| **PACIFIC** | **Pred endotype 1** | **Pred endotype 2** | **Pred endotype 3** | **Pred endotype 4** | **Overall** |
| --- | --- | --- | --- | --- | --- |
|  | **(N=122)** | **(N=33)** | **(N=13)** | **(N=23)** | **(N=191)** |
| ***Coronary CT angiography*** | | |  |  |  |
| **High risk plaque n (%)** | 22 (18.0) | 9 (27.3) | 3 (23.1) | 8 (34.8) | 42 (22.0) |
| **Absence of CHD n (%)** | 23 (18.9) | 2 (6.1) | 0 (0.0) | 0 (0.0) | 25 (13.1) |

CHD: coronary heart disease

Absence of CHD refers to coronary calcium score of zero and the absence of coronary plaques from CCTA

High-risk-risk plaque was defined as a coronary lesion with more than two adverse plaque characteristics

## **Supplementary Figures**

### **Figure S1.** Flow chart displaying inclusion and exclusion criteria applied in the present study


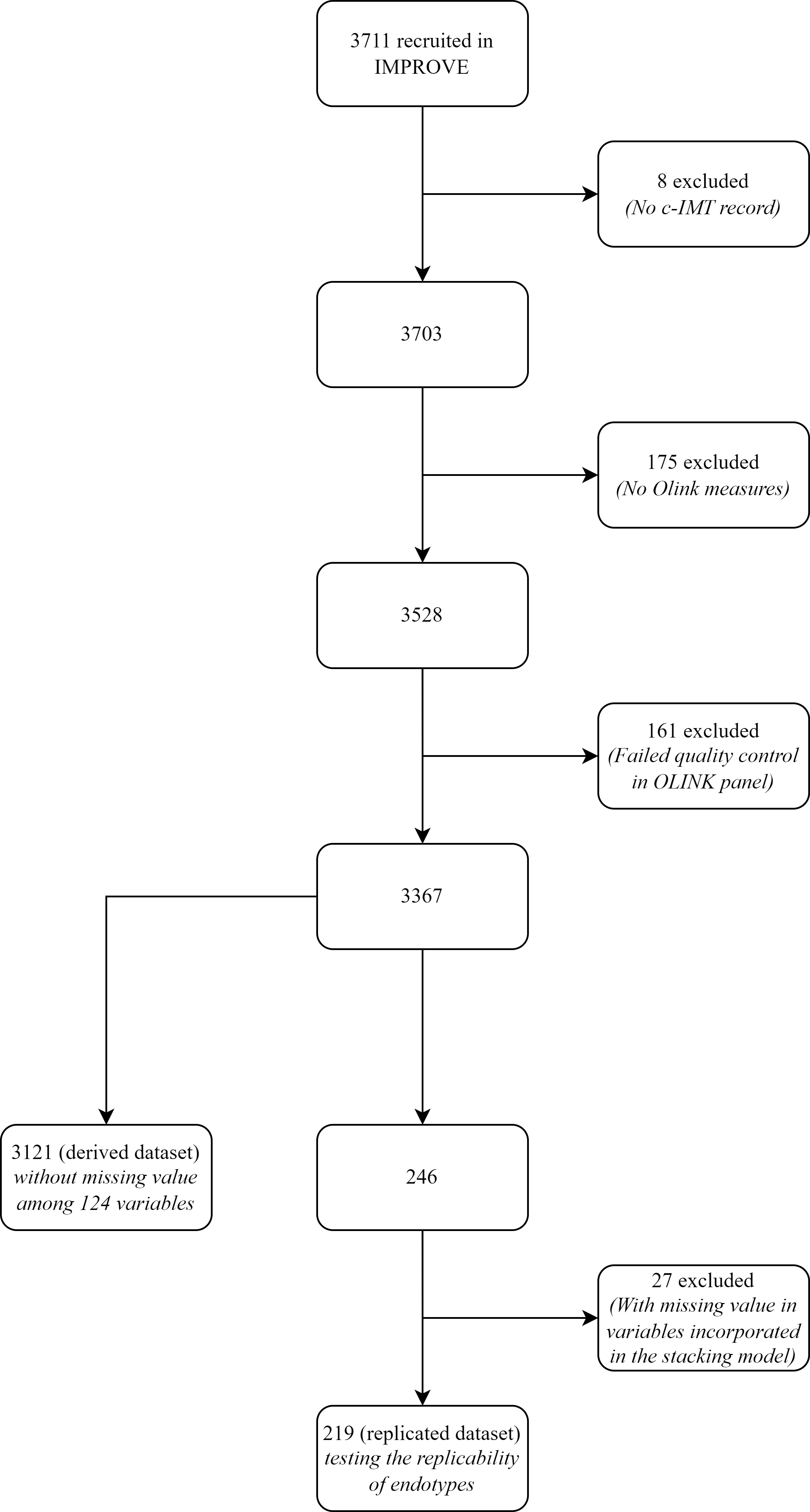


C-IMT refers to carotid intima-media thickness.

### **Figure S2.** Overview of the analytical approach


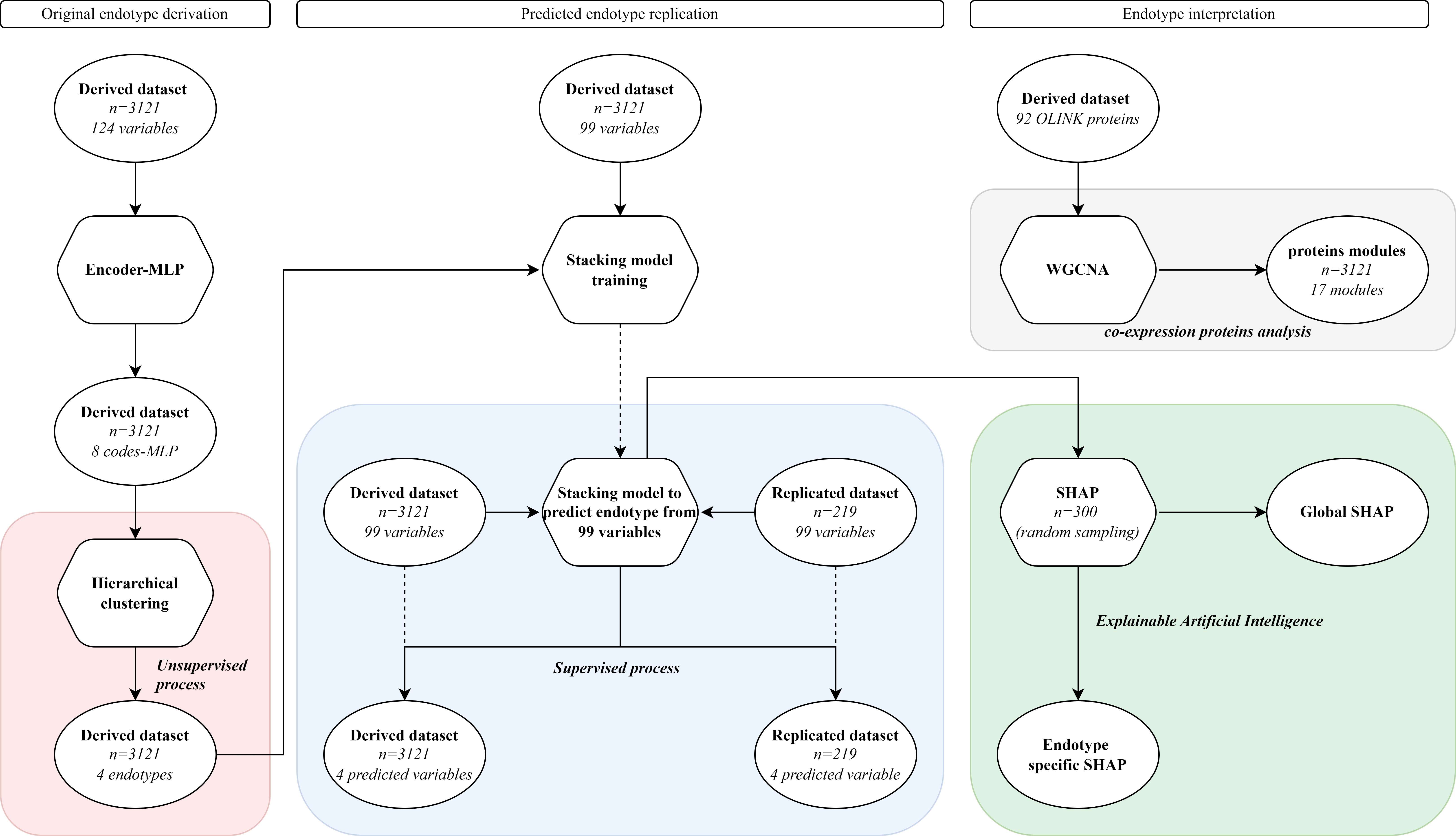


Hexagonal shapes include description, comparison and/or modelling. Oval shapes include output data from the different statistical models.

Definition of derived and replicated dataset are reported in Supplementary Figure I.

C-IMT: carotid intima-media thickness;

### **Figure S3.** Encoder-MLP structure display for c-IMT feature engineering


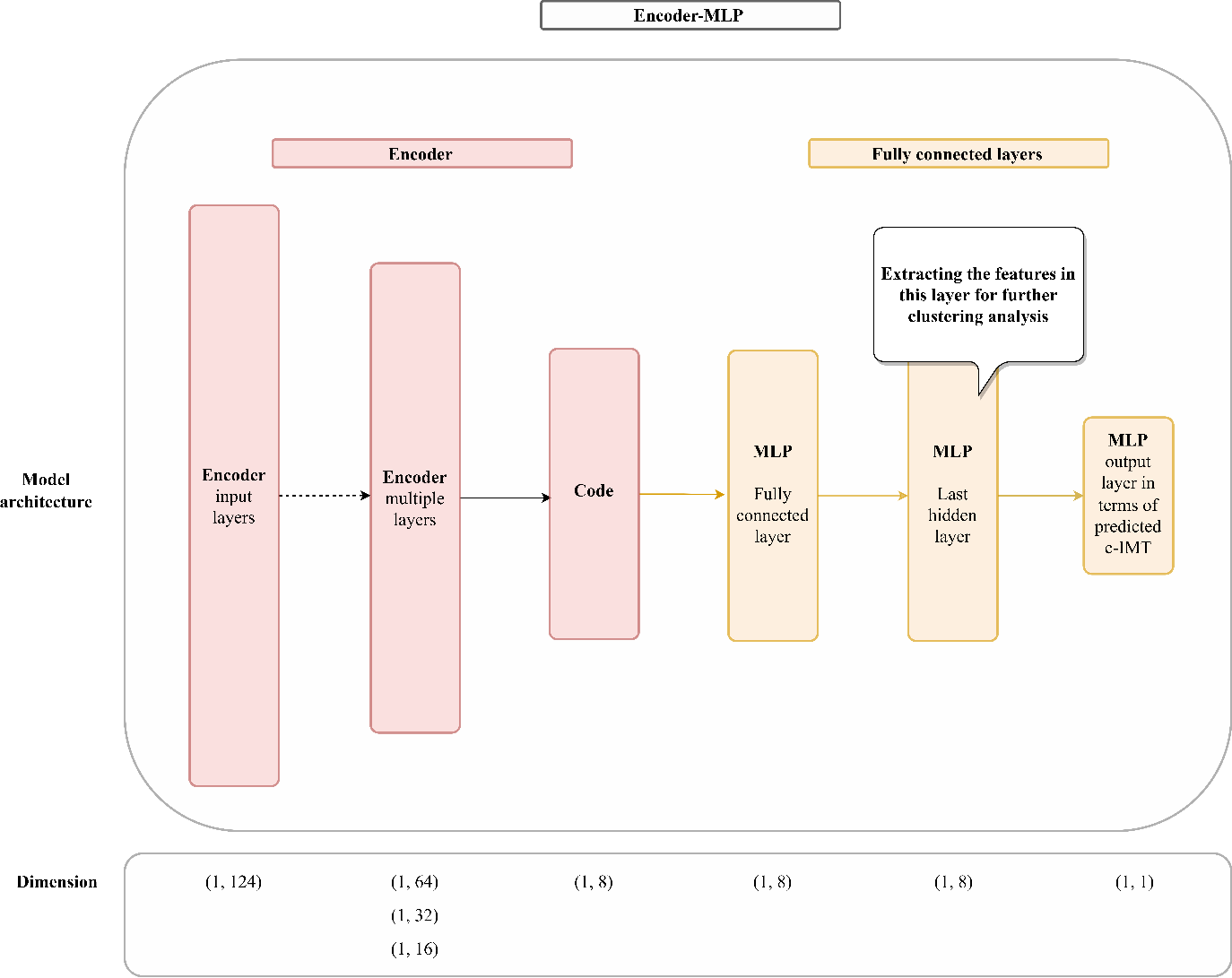


The model architecture of Encoder-MLP consists of two sequential modules, an Encoder and fully connected layers.

In the Encoder modules, a 5-layers encoder (indicated as dimension in the lower panel of the figure, 124-64-32-16-8) reduces the dimension of the 124 variables into 8 codes, directly linked to two fully connected layers with 8 computation units (yellow bars in the upper panels). The Encoder-MLP with c-IMT_mean-max_ as output layer filters sub-clinical atherosclerosis related information.

After input the 124 variables, one can extract the processed information in this last hidden layer of the Encoder-MLP (codes-MLP) for downstream cluster analysis.

### **Figure S4.** Bar plot showing the number of clusters supported by 20 metrics. Six metrics identified 4 as the optimal number of clusters


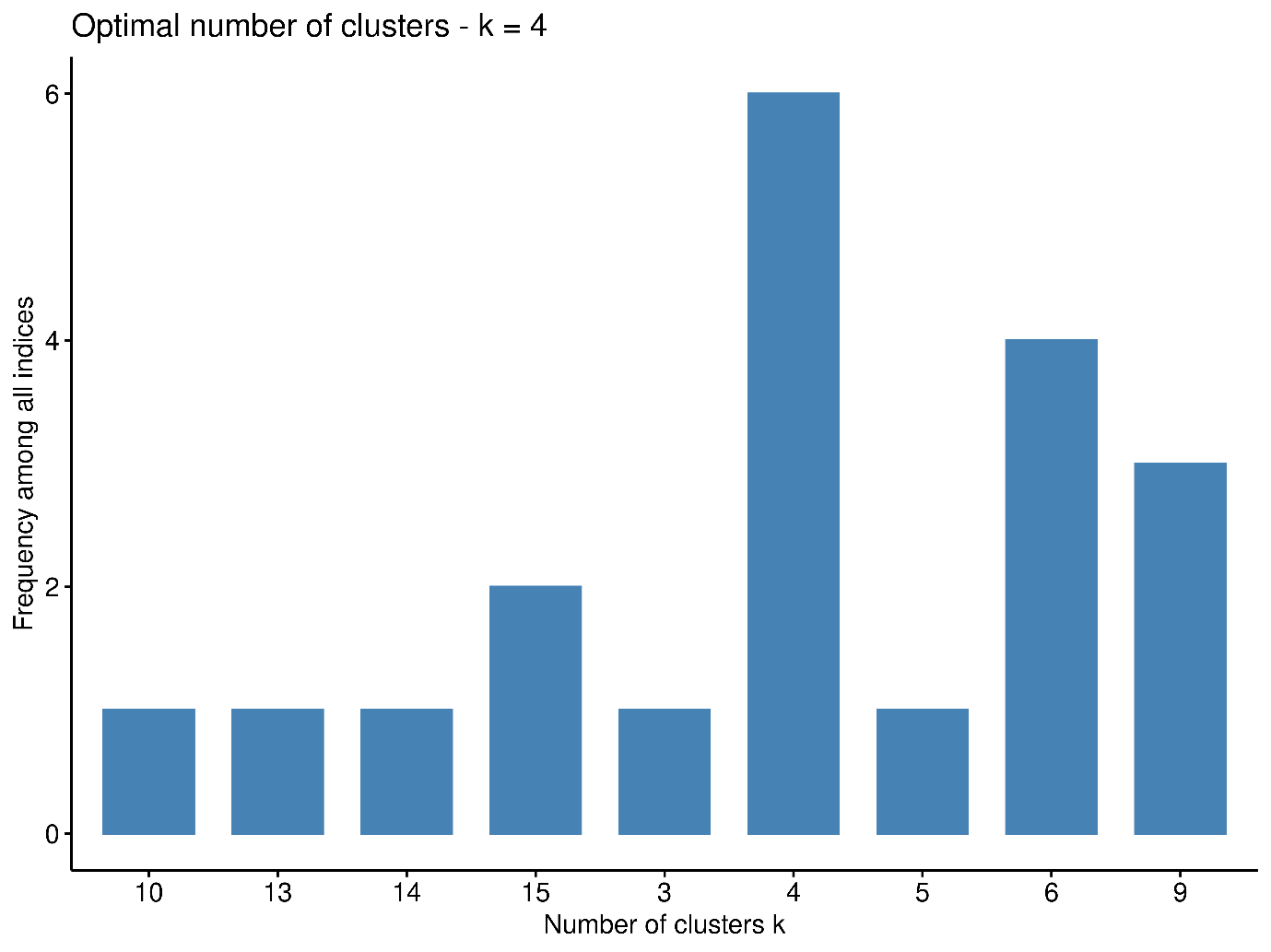


Each bar represents the number of statistics (Y axis) supporting the choice of number of clusters. A total of 20 statistics were used, 6 of them support 4 as the ideal number of clusters. As an example, 1 statistic support 10 clusters as the optimal cluster number and 2 statistics support 15 clusters as optimal number of clusters. Based on the majority rules, we chose to define 4 clusters for further analysis.

### **Figure S5.** Atherosclerosis related information filtration in different layer of the Encoder-MLP


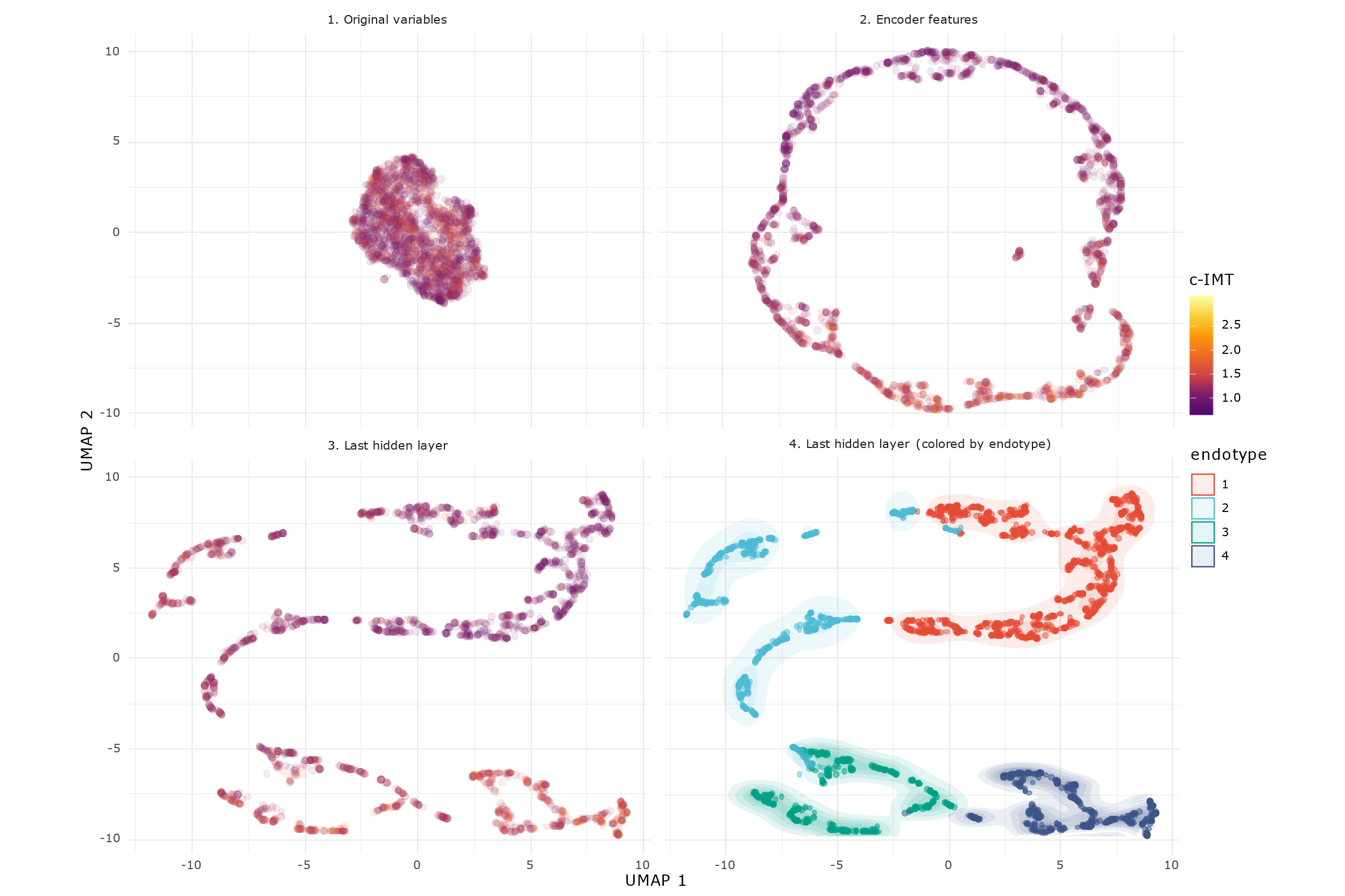


UMAP: Uniform Manifold Approximation and Projection. UMAP1 and UMAP2 refers to two dimensions after UMAP transformation.

The set of plots shows the atherosclerosis-related information filtration by Encoder-MLP. The upper left panel shows the original distribution of the 124 variables. The upper right panel shows the variables distribution in the mid of Encoder-MLP processing of the data. The lower left panel represents the variables distribution in the last hidden layer of the Encoder-MLP. The lower right panel shows the results of the hierarchical clustering that identifies four c-IMT endotypes.

For UMAP tuning, a was set as 1.75 and b was set as 0.75.

### **Figure S6.** Feature engineering using a random number with a comparable distribution to the c-IMT_mean-max_ to extract atherosclerosis related features from the 124 variables





UMAP: Uniform Manifold Approximation and Projection. UMAP1 and UMAP2 two dimension after UMAP transformation.

The set of plots shows the random-number-related information filtration by Encoder-MLP. The upper left panel shows the original distribution of the 124 variables. The upper right panel shows the variables distribution in the mid of Encoder-MLP processing of the data. The lower left panel represents the variables distribution in the last hidden layer of the Encoder-MLP.

For UMAP tuning, a was set as 1.75 and b was set as 0.75.

The random number has the same mean, standard deviation as c-IMT_mean-max_.

### **Figure S7.** Chord diagram showing the prediction performance of stacking model





The chord diagrams show the pair-wise agreement evaluation between original endotype and Pred endotype (PACIFIC), between original endotype and Pred endotype (PIVUS), and between pred endotype (PIVUS) and pred endotype (PACIFIC). The systematic evaluation of endotype prediction model for external validation using Cohen’s weighted Kappa were performed, with the Cohen’s weighted Kappa value of 0.89.

Reference cut-off of kappa value: (1) less than 0: no agreement; (2) 0.01–0.20: slight agreement; (3) 0.21–0.40: fair agreement; (4) 0.41– 0.60: moderate agreement; (5) 0.61–0.80: substantial agreement; (6) 0.81–1.00: perfect agreement

Of note, hierarchical clustering is sensitive to background noise. This implies that misclassification of some study participants is expected. In particular, we observed the largest misclassification might occur between neighbored endotype, such as misclassification from endotype 1 to 2, or 3 to 4.

Endotype refer to the endotype from hierarchical clustering.

Pred endotype (PACIFIC) refer to the predicted endotype using the model for PACIFIC.

Pred endotype (PIVUS) refer to the predicted endotype using the model for PIVUS.

### **Figure S8.** Global SHAP value estimates the predictive value of the 20 most common variables within each endotype.


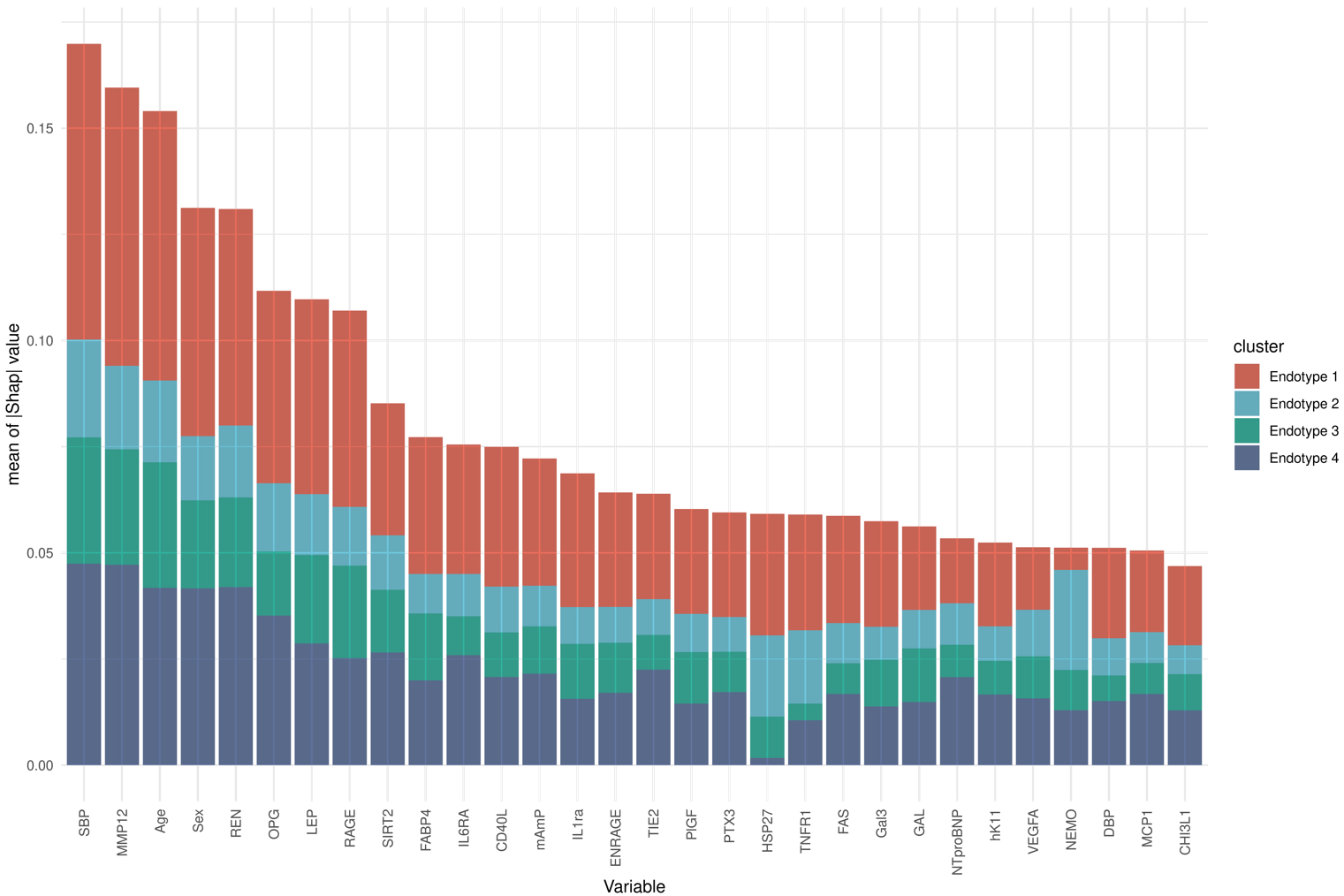


The bar graph shows the mean of global |SHAP| value for the 20 most relevant variables identified in the stacking model for endotypes 1-4. Each endotype is labelled with a different colour (red: endotype 1, turquoise: endotype 2; green: endotype 3 and purple: endotype 4). The figure provides information on the endotype specific and on the mean of global |SHAP| value for each variable. The variables are listed on the X axis in descending order according to their value in global endotype prediction. SHAP value was estimated from 300 random samples in derived dataset (n=3121). As an example, SBP is the most relevant variable for endotype 1 and the variable with the highest global impact on the endotype prediction; NEMO is the most relevant variable for endotype 2, but globally has a relatively low impact on the prediction of the outcome

### **Figure S9**. Weighted co-expression network analysis to define protein co-expression modules and relative concentration of each biomarker in the 4 endotypes.


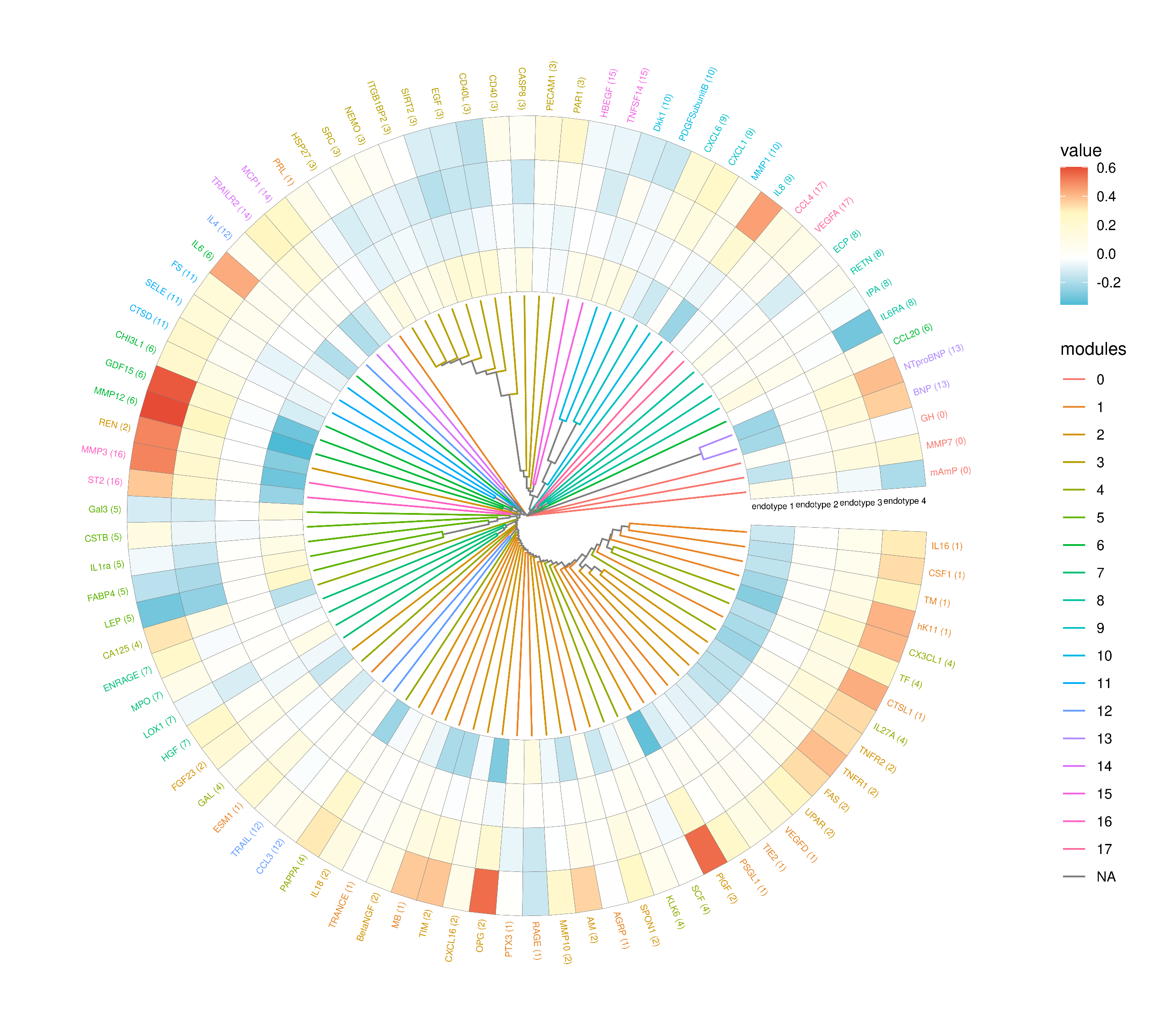


The figure shows a dendrogram where each color represents a protein module, from 1 to 17. Each module consists of co-expressed proteins. Only proteins in modules 1, 2, 3, 5, 6, and 8 are represented in the endotypes Modules 0 proteins cannot be assigned to any module. For each protein in heatmap, we labelled the protein modules within brackets.

The relative circulating levels of each protein (Z-scoresample) are graded according to a color scale as shown in the upper right corner. Z-scoresample was calculated by the formula: (sample value-mean)/standard deviation. As circulating levels were standardized, the mean of each protein equals to zero (white color). Z-scoresample lower than zero are represent in light blue and Z-scoresample over the zero in red. The inner circle corresponds to endotype 1 and the outer to endotype 4. In the middle are endotype 2 and endotype 3.

As an example, for MMP12 in module 6, standardized circulating levels were lower than zero in endotype 1, equal to zero in endotype 2, slightly higher than zero in endotype 3 and largely higher than zero in endotype 4.

We provide the zoom-in figure for proteins in modules 1, 2, 3, 5, 6, and 8 as Figure 2 in main text.
